# Supplementary material for: miRNA Clusters with Up-Regulated Expression in Colorectal Cancer
Source: Cancers (Basel). 2021 Jun 14;13(12):2979. doi: 10.3390/cancers13122979 (PMC8232258; doi:10.3390/cancers13122979)
Supplement: Supplementary file 1 [file cancers-13-02979-s001.zip › Supplementary tables 1-6 and S8.pdf]

Supplementary material S1: List of included studies for selection up-regulated miRNA clusters in CRC.

First author	Year	Ref.	Country/ Region	Tumour type	Control samples	Method of analysis	Upregulated miRNAs	Normalization
Motoyama	2009	[270]	Japan	CRC	PAT	microarray	21	RNU6-6P
Ng	2009	[97]	Hong Kong	CRC	PAT	array	14	RNU6-1
Arndt	2009	[271]	Australia	CRC	NT	array	22	quantile normalization
Wang	2010	[272]	China	CC	PAT	microarray	12	RNU6-1
Huang	2011	[273]	Shangai	CRC	PAT	microarray	26	RNU6-1
Fu	2012	[274]	China	CRC	PAT	microarray	16	quantile scaling algorithm
Hamfjord	2012	[275]	Norway	CAD	PAT	sequencing	24	SNORD48
Chen	2012	[276]	China	CRC	PAT	array	32	RNU6-6P
Li	2012	[277]	China	RC	PAT	miRNA chip	65	RNU6-6P
Mosakhani	2012	[278]	Finland	CRC	NT	microarray	7	RNU6-1
Nishida	2012	[279]	Japan	CRC	NT	microarray	127	LOWESS method
Pizzini	2013	[3]	Italy	CRC	PAT	microarray	29	SNORD44
Chen	2013	[280]	Taiwan	CRC	PAT	array	5	RNU6-1
Ak	2014	[281]	Turkey	CRC	NT	qPCR	1	SNORD 44, SNORD47, SNORD 48
Li	2014	[282]	USA	CRC	PAT	microarray	49	miR-191
Fukushima	2015	[283]	Japan	CRC	PAT	array	5	RNU6-1
Mo	2015	[284]	Korea	CRC	PAT	microarray	29	SNORD48, RNA5-8S5
Peng	2015	[285]	China	CRC	PAT	qPCR	12	RNU6-1
Rotelli	2015	[114]	Italy	CRC	NT	qPCR	11	RNU6-6P, miR-16-3p
Tanoglu	2015	[286]	Turkey	CC	PAT	qPCR	4	RNU6-6P
Vishnubalaji	2015	[2]	Saudi Arabia	CC	PAT	microarray	42	SNORD44, SNORD48
Wu	2015	[287]	China	CRC	PAT	qPCR	3	RNU6-6P, SNORD44, SNORD48, SNORD49A
Neerincx	2015	[288]	Netherlands	CRC	NT	NGS	121	edgeRs TMM method
Meng	2015	[289]	China	CRC	PAT	array	11	NS
Kara	2015	[290]	Turkey	CRC	NT	array	19	SNORD61, SNORD68, SNORD72, SNORD95
Chen	2015	[263]	China	CRC	PAT	qPCR	4	RNU6-6P
Ozcan	2016	[291]	Turkey	CRC	NT	array	20	SNORD61, SNORD68, SNORD96A, RNU6-2
Pelossof	2016	[292]	USA	RC	PAT	sequencing	39	Quantile normalization
Sun	2016	[293]	USA	CRC	PAT	sequencing	12	SNORD47
Zekri	2016	[294]	Egypt	CRC	NT	qPCR	4	SNORD68
Li	2017	[295]	China	CRC	PAT	miRNA microarray	17	RNU-6
Nagy	2017	[296]	Hungary	CRC	NT	microarray	6	Hsa-miR-423-5
Wu	2017	[297]	-	CAD	NT	sequencing	99	Log10

Du	2018	[298]	China	CRC	PAT	miRNA array	182	RMA algorithm
Pellatt	2018	[299]	USA	CRC	PAT	microarray	36	Scaling factor based on median expression
Slattery	2018	[140]	USA	CRC	NT	microarray	22	Scaling factor based on median expression
Wu	2018	[255]	USA	CRC	NT	miRNA sequencing	34	Localized smoothing algorithm
Zhang	2018	[300]	China	CRC	PAT	miRNA microarray	1	NS
Brînzan	2019	[301]	Romania	CAD	PAT	qPCR	5	miR-26b, miR-92N
Li	2019	[302]	China	CRC, CAD	NT	miRNA sequencing	34	NS
Zhang	2019	[303]	China	CRC	NT	qPCR	4	RNU6-6P
Gungormez	2019	[304]	Turkey	CC	PAT	array	3	NS
Hao	2019	[305]	China, Italy, USA	CRC	PAT	miRNA array	8	RNU6-1
Duran-Sanchon	2020	[119]	Spain	CRC	PAT	NGS	21	Cyclic-loess method
Wei	2020	[306]	China	CC, RC	PAT	miRNA microarray	4	RNU6-1
Milanesi	2020	[307]	Romania	CRC	NT	array	1	SNORD61, SNORD95, SNORD96A
Zhou	2020	[308]	China	CRC	NT	qPCR	226	RNU6-1

Tumour type – CRC (colorectal cancer); CAD (colorectal adenocarcinoma); RC (rectal cancer). miRNA expression in tumour in referred studies was compared to paired adjacent tissue (PAT) or to normal tissue (NT). Method of analysis – NGS – next gene sequencing; qPCR - quantitative real-time PCR. Normalization – NS – not specified; RNA5-8S5 - RNA, 5.8S ribosomal 5; RNU6-1 - RNA, U6 small nuclear 1; RNU6-2 - RNA, U6 small nuclear 2; RNU6-6P - RNA, U6 small nuclear 6, pseudogene; SNORD44 - small nucleolar RNA, C/D box 44; SNORD47 - small nucleolar RNA, C/D box 47; SNORD48 - small nucleolar RNA, C/D box 48; SNORD49A - small nucleolar RNA, C/D box 49A; SNORD61 - small nucleolar RNA, C/D box 61; SNORD68 - small nucleolar RNA, C/D box 68; SNORD72 - small nucleolar RNA, C/D box 72; SNORD95 - small nucleolar RNA, C/D box 95; SNORD96A - small nucleolar RNA, C/D box 96A. Ref. – refference.

Supplementary material S2: Up- or down-regulation of the analysed miRNA clusters levels in tumour tissue and circulation of patients with CRC

Cluster	miRNA	Expression in tumour tissue	Levels in serum	Levels in plasma
miR-106a/18b/20b/19b-2/92a-2/363	**miR-106a**	↑ [3,96,277,281,284,286,293,298,308–311,97,312,114,130,131,134,158,255,271]	↑ [104,312]<br>↓ [313]	↑ [133]
	**miR-18b**	↑ [2,3,298,309,314,270,272,273,277,279,282,295,296]		↑ [303]<br>↓ [314]
	**miR-20b**	↑ [2,3,140,279,284,299,309]<br>↓ [208,276,308,315]		↑ [316]
	**miR-19b**	↑[114,115,292,299,302,308,317,140,158,255,271,274,282,284,288]<br>↓ [318]		
	**miR-92a**	↑ [97,114,270,277,279,282,288,289,292,298,299,308,115,319–321,119,138–140,158,202,255]<br>↓ [318]	↑ [129,136,154,322,323]<br>↓ [324]	↑ [99,325,326]
	**miR-363**	↓ [144,275,276,282,308,327]	↓ [328]	
miR-106b/93/25	**miR-106b**	↑ [2,3,284,288,292,298,299,302,308,329,330,97,119,255,271,272,274,277,279]<br>↓[234,288,308,331,332]	↑ [330]	↑ [325]
	**miR-93**	↑ [140,202,308,333,271,274,279,282,284,288,289,298]<br>↓ [81,145,147,222]	↓ [324]	
	**miR-25**	↑[3,119,282,284,288,298,299,308,334,335,140,148,149,202,271,274,277,279]<br>↓ [336]	↑ [335,337]<br>↓ [324]	
miR-17/18a/19a/20a/19b-1/92a-1	**miR-17**	↑ [2,3,270,271,273,274,276,277,279,282,284,288,97,289,290,292–294,298,299,303,308,309,114,314,334,338,339,115,119,140,158,202,255,263]<br>↓ [3,318]	↑ [104,294]	↑ [97,303,326]<br>↓ [314]
	**miR-18a**	↑[2,3,270–273,277,279,280,282,288,292,57,296,298,302,303,309,323,338,97,114,115,117,119,153,255,305]<br>↓ [318,340]	↑ [323]	↑[303,325]
	**miR-19a**	↑ [2,3,277,279,284,288,291,292,294,302,308,309,114,341–343,115,132,255,271–274]<br>↓ [318]	↑[154,294,322,344–347]	
	**miR-20a**	↑ [2,3,140,155,158,270,272–274,277,279,282,29,284,288,289,291,292,299,308,309,314,323,65,338,348–351,97,114,115,119,130,134,305,263]<br>↓ [318]	↑ [294,323,344,350]<br>↓ [352]	↑[133,185,325,348]<br>↓ [314]
	**miR-19b**	↑[2,114,299,302,308,317,115,140,158,255,274,279,284,292]<br>↓ [318]	↑ [104]	

	**miR-92a**	↑ [3,97,255,270,277,279,282,288,289,292,298,299,114, 308,319–321,115,119,138–140,158,202] ↓ [318]	↑[129,136,154,322,323, 353] ↓ [324,354]	↑ [97,99,325,326]
miR-181a-1/181b-1 miR-181a-2/181b-2	**miR-181a**	↑ [29,158–160,279,291,308,355] ↓ [42,82]	↑ [104]	↑ [303]
	**miR-181b**	↑ [134,171,271,279,291,298,308,356] ↓ [357]	↑ [358] ↓ [354]	↑ [97]
miR-181c/181d	**miR-181c**	↑ [279,282,284,288,289,291]		↑ [97]
	**miR-181d**	↑[3,34,298,359,158,161,270,277,279,288,291,292]		↑ [97]
miR-183/ 96/182	**miR-183**	↑ [2,3,276,277,279,282,288,291,292,301,302,360,158, 361,362,162,164,202,255,270,271,273,305,263]		↑ [97]
	**miR-96**	↑[2,119,276,277,280,282,283,287,288,290,292,308,158, 309,323,363,165,220,255,271,273–275]	↑ [323]	
	**miR-182**	↑ [2,3,271,273,276,277,282,285,287–289,292,29,293, 298,301,308,364,365,100,119,158,168,169,255,270] ↓ [366]	↑ [105]	↑ [100,185,367]
miR-191/ 425	**miR-191**	↑ [289,298,308,368,369]		
	**miR-425**	↑[2,140,279,282,298,299,302,308,346,370]	↑ [337,346]	
miR-200c/ 141	**miR-200c**	↑ [158,171,373,172,173,177,255,291,308,371,372] ↓ [348,374–376]	↑ [104,373,377] ↓ [378]	↑[97] ↓ [348]
	**miR-141**	↑ [114,177,379–381,255,277,282,285,289,301,308,371] ↓ [78,179,382]	↑ [377,383] ↓ [382]	
miR-203a/203b	**miR-203**	↑ [130,158,271,277,282]	↓ [354]	
	**miR-203a**	↑ [2,119,140,180,202,255,290,302,308,384] ↓ [58,181,238,385–387]	↑ [180,378]	↑ [97]
	**miR-203b**	↓ [72,308]	↓ [388]	
miR-222/ 221	**miR-222**	↑ [288–290,308] ↓ [376]		↑ [97,103]
	**miR-221**	↑[2,97,292,299,302,308,117,119,140,198,273,276,279, 282]	↑ [389,390]	↑ [103]
miR-23a/ 27a/ 24-2	**miR-23a**	↑ [158,279,394,288,295,298,299,308,391–393]	↑ [358] ↓ [391,395]	↑ [103]
	**miR-27a**	↑[158,184,298,394,396,186,274,275,277,284,290,292, 295] ↓ [376,397]	↑ [124]	↑ [103]
	**miR-24**	↑ [119,158,277–279,292,299,308,394] ↓ [188,275,341]	↓ [398]	↓ [399,400]
miR-29b-1/ 29a	**miR-29b**	↑ [3,29,302,140,158,271,274,277,282,290,295] ↓ [64,101,189,376,401]	↓ [402]	↓ [101]
	**miR-29a**	↑ [192,271,277,284,290,292,323,403] ↓ [404]	↑[323,345,405] ↓ [389]	↑ [99,325]
miR-301b/ 130b	**miR-301b**	↑ [272,275,288,308]		

	**miR-130b**	↑[2,119,308,406,194,271,279,282,285,292,298,299]	↑ [337]
miR-452/ -224	**miR-452**	↑ [158,195,255,276,277,279,284,288,308]<br>↓ [196]	↓ [388]
	**miR-224**	↑[2,3,273,274,279,282–284,288,292,293,298,97,305,308<br>,323,407–409,197,198,233,255,270–272]<br>↓ [410]	↑ [104]<br>↓ [388]

Supplementary material S3: Analysis of miRNA expression with clinicopathological characteristics.

cluster	miRNA	advanced TNM stage	Presence of metastasis
miR-106a/18b/20b/19b-2/92a-2/363	**miR-106a**		↑ TU [411]
	**miR-18b**		↑TU [295]
	**miR-363**	↓ S [328]	↓ S [328]<br>↓ TU [144,327]
miR-106b/93/25	**miR-106b**	↑ S [330]	↓ TU [74,234,332]
	**miR-93**		↑ TU [145]
	**miR-25**	↑ TU [148]	↑ TU [148]; S [335,337]
miR-17/18a/19a/20a/19b-1/92a-1	**miR-17**		↑ M [412]; P [326]; TU [321]<br>↓ TU [413]
	**miR-18a**		↑ S [390]
	**miR-19a**	↑ S [344]; TU [342]	↑ TU [236,342] S [343,344]<br>↓P [414]
	**miR-20a**	↑ S [344]; TU [415]	↑S [344]; TU [155,415]
	**miR-19b**		↑M [156]; TU [317]
	**miR-92a**	↑ TU[137,138]<br>↑ S [353]	↑ TU [137,139,279,319,321,416]; P [326];<br>S [319,353]
miR-181a-1/181b-1<br>***miR-181a-2/181b-2***	**miR-181a**	↑ TU [159]	↑ TU [160,176]
miR-181c/181d	**miR-181d**		↑ TU [34]
miR-183/96/182	**miR-183**	↑ TU[162,370]; P [360]	↑ TU [162]; P [360]
	**miR-96**	↑ TU [165]	↑ TU [165]
	**miR-182**	↑ TU [169,196,301]	↑ TU [168,169,176]
miR-191/425	**miR-191**	↑ TU [417]	↑ TU [417]<br>↓TU [170]
	**miR-425**		↑ S [337]
miR-200c/141	**miR-200c**	↑TU [177,373]; S [173]<br>↓TU [418]	↑TU [173,373,419]; S [173,371]<br>↓TU [176,418,420]
	**miR-141**	↑ TU [177]; P [166,178]<br>↓ TU [179]	↑ TU [36]; S [371,421]<br>↓ TU [179]
miR-203a/203b	**miR-203a**	↑ S [180]<br>↓ TU [422]	↑ S [180]<br>↓ TU [181,386]
miR-222/221	**miR-221**	↓ TU [182]	↓TU [182]
miR-23a/27a/24-2	**miR-23a**	↑ BL [391]	
	**miR-27a**	↑ TU [186]	↑ TU [183,184,423]; P [185]
	**miR-24**	↓ TU [188,341]	↓ TU [188,341]
miR-29b/ 29a	**miR-29b**	↓S [402]	
	**miR-29a**	↑S [424]<br>↓ TU [64]	↑ TU[190,425]<br>↑ S [190]<br>↓ TU [64,176,401]

miR-301b/ 130b	**miR-130b**		↑ TU [194]
	**miR-224**	↓ TU [182]	↑ LM [407,409]; TU [198,408]<br>↓ LNM [409]; TU [182]

↑ depicts increased expression/levels associated with advanced TNM stage or metastasis, respectively ↓ decreased expression/levels. Source for measurement of miRNA expression is bolded – TU (tumor tissue); P (plasma); S (serum); LM (liver metastasis); BL (blood); LNM (lymph node metastases); M - metastasis

Supplementary material S4: Alphabetical list of target genes of up-regulated miRNA clusters.

Gene symbol	Gene name	Function	miRNA	Cluster	Reference
ABCA1	ATP binding cassette subfamily A member 1	TS	miR-183-5p	miR-183/96/182	[426]
ABCF1	ATP-binding cassette, subfamily F, member 1	TS	miR-23a-3p	miR-23a/27a/24-2	[427]
ADAM9	ADAM metallopeptidase domain 9	O	miR-20b-5p	miR-106a/363	[208]
AFDN	Afadin, Adherens junction formation factor	TS	miR-183-3p	miR-183/96/182	[428]
AGER	Advanced glycosylation end-product specific receptor	TS	miR-182	miR-183/96/182	[48]
AKAP12	A kinase anchor protein 12	TS	miR-183-5p	miR-183/96/182	[370]
AKT2	AKT serine/threonine kinase 2	O	miR-203a-3p	miR-203a/203b	[429]
AMER1	APC membrane recruitment protein 1	TS	miR-20a	miR-17/92a-1	[310]
			miR-106a-5p	miR-106a/363	[310]]
ANGPTL8	Angiopoietin like 8	O	miR-25	miR-106b/25/93	[430]
APAF1	Apoptotic peptidase activating factor 1	TS	miR-23a-3p	miR-23a/27a/24-2	[392]
AQP8	Aquaporin 8	TS	miR-92a-5p	miR-106a/363<br>miR-17/92a-1	[431]
ARID4B	AT-rich interaction domain 4B	TS	miR-93-3p	miR-106b/25/93	[55]
ATG12	Autophagy related 12	O	miR-93-5p	miR-106b/25/93	[66]
ATG16L1	Autophagy Related 16 Like 1	TS	miR-106b-5p<br>miR-93-5p	miR-106b/25/93	[432]
ATG5	Autophagy related 5	TS	miR-20a-5p	miR-17/92a-1	[433]
			miR-183-5p	miR-183/96/182	[164]
ATG7	Autophagy related 7	TS	miR-106a-5p	miR-106a/363	[131]
ATM	ATM serine/threonine kinase	TS	miR-18a-5p	miR-17/92a-1	[153]
			miR-203a-3p	miR-203a/203b	[232]
ATXN3	Ataxin 3	TS	miR-25-3p	miR-106b/25/93	[149]
BCL2L1	BCL2 like 1	O	miR-203b-3p	miR-203a/203b	[72]
BCL2L11	BCL2 like 11	TS	miR-92a-3p	miR-106a/363<br>miR-17/92a-1	[434]
BCL9L	BCL9 like	O	miR-29b-3p	miR-29b-1/29a	[435]
BID	BH3 interacting domain death agonist	TS	miR-20a-5p	miR-17/92a-1	[350]
BMI1	BMI1 proto-oncogene, polycomb ring finger	O	miR-200c-3p	miR-200c/141	[241]
BNIP2	BCL2 interacting protein 2	TS	miR-20a-5p	miR-17/92a-1	[436]
BTG1	BTG anti-proliferation factor 1	TS	miR-27a-3p	miR-23a/27a/24-2	[437]
BTG3	BTG anti-proliferation factor 3	TS	miR-17-5p	miR-17/92a-1	[68]
CEBPB	CCAAT enhancer binding protein beta	TS	miR-191-5p	miR-191/425	[369]

CALR	Calreticulin	TS	miR-27a-3p	miR-23a/27a/24-2	[438]
CCND1	Cyclin D1	O	miR-93-5p	miR-106b/25/93	[222]
			miR-18a-5p	miR-17/92a-1	[224]
			miR-96-5p	miR-183/96/182	[167]
CCND2	Cyclin D2	O	miR-141-3p	miR-200c/141	[375]
CD274	CD274 molecule	O	miR-93-5p	miR-106b/25/93	[147]
CDC42	Cell division cycle 42	O	miR-18a-5p	miR-17/92a-1	[224]
CDH1	Cadherin 1	TS	miR-224-5p	miR-452/224	[198]
CDK19	Cyclin dependent kinase 19	O	miR-18a-5p	miR-17/92a-1	[439]
CDK2	Cyclin Dependent Kinase 2	O	miR-200c-3p	miR-200c/141	[43]
CDKN1A	Cyclin dependent kinase inhibitor 1A	TS	miR-106b-5p	miR-106b/25/93	[234]
			miR-20a-5p	miR-17/92a-1	[235]
			miR-224-5p	miR-452/224	[233]
CDKN2B	Cyclin dependent kinase inhibitor 2B	TS	miR-18b-5p	miR-106a/363	[295]
CDS2	CDP-diacylglycerol synthase 2	O	miR-224-5p	miR-452/224	[440]
CLU	Clusterin	TS	miR-17	miR-17/92a-1	[33]
CPEB4	Cytoplasmic polyadenylation element-binding 4	O	miR-203a-3p	miR-203a/203b	[226]
CREB1	cAMP responsive element binding protein 1	O	miR-203a-3p	miR-203a/203b	[54]
CRY2	Cryptochrome circadian regulator 2	TS	miR-181d-5p	miR-181c/181d	[34]
CSE1L	Chromosomal segregation 1 like	O	miR-203a-3p	miR-203a/203b	[49]
CTNNB1	Catenin Beta 1	O	miR-93-5p	miR-106b/25/93	[222]
			miR-181a-5p	miR-181a-1/181b-1<br>miR-181a-2/181b-2	[56]
			miR-203a-3p	miR-203a/203b	[67]
			miR-24-5p	miR-23a/27a/24-2	[441]
CTNND1	Catenin delta 1	TS	miR-425-5p	miR-191/425	[442]
CTSA	Cathepsin A	O	miR-106b-5p	miR-106b/25/93	[332]
CXCL8	C-X-C motif chemokine ligand 8	O	miR-20a-5p	miR-17/92a-1	[443]
CYP7B1	Cytochrome P450, family 7, subfamily B, member 1	O	miR-17-5p	miR-17/92a-1	[368]
DAB2IP	DAB2 interacting protein	TS	miR-182-5p	miR-183/96/182	[105]
DHFR	Dihydrofolate reductase	O	miR-24-3p	miR-23a/27a/24-2	[444]
DKK3	Dickkopf WNT signalling pathway inhibitor 3	TS	miR-92a-3p	miR-106a/363<br>miR-17/92a-1	[210]
DLC1	DLC1 Rho GTPase activating protein	TS	miR-106b-5p<br>miR-106b-3p	miR-106b/25/93	[329,330]
			miR-141-3p	miR-200c/141	[381]
DND1	DND microRNA-mediated repression inhibitor 1	O	miR-24-3p	miR-23a/27a/24-2	[445]
DNMT3B	DNA methyltransferase 3 beta	O	miR-203a-3p	miR-203a/203b	[385]

DUSP2	Dual-specificity phosphatase 2	TS	miR-106a-5p	miR-106a/363	[311]
E2F5	E2F transcription factor 5	O	miR-181a-5p	miR-181a-1/181b-1<br>miR-181a-2/181b-2	[446]
EGFR	Epidermal growth factor receptor	O	miR-141-3p	miR-200c/141	[447]]
EIF5A2	Eukaryotic initiation factor 5A2	O	miR-203a-3p	miR-203a/203b	[181]
ETS1	ETS proto-oncogene 1, transcription Factor	O	miR-200c-3p	miR-200c/141	[36]
EZH2	Enhancer of zeste 2 polycomb repressive complex 2 subunit	O	miR-363-3p	miR-106a/363	[73]]
F2RL3	F2R like thrombin or trypsin receptor 3	TS	miR-17-3p	miR-17/92a-1	[339]
FAM172A	Family with sequence similarity 172 member A	TS	miR-27a-3p	miR-23a/27a/24-2	[186]
FBXL3	F-box and leucine rich repeat protein 3	TS	miR-181d-5p	miR-181c/181d	[34]
FBXW7	F-Box and WD repeat domain containing 7	TS	miR-92a-3p	miR-106a/363<br>miR-17/92a-1	[319]
			miR-182-5p	miR-183/96/182	[448]
FLT1	Fms related receptor tyrosine kinase 1	O	miR-200c-3p	miR-200c/141	[36]
FOXA1	Forkhead box A1	TS	miR-93-5p	miR-106b/25/93	[333]]
FOXF2	Forkhead box F2	TS	miR-19a-3p	miR-17/92a-1	[347]
			miR-182-5p	miR-183/96/182	[449]
FOXJ2	Forkhead Box J2	O	miR-20a-5p	miR-17/92a-1	[450]
FOXO1	Forkhead box protein O1	TS	miR-96-5p<br>miR-182-5p	miR-183/96/182	[220]
FOXO3a	Forkhead box protein O3a	TS	miR-96-5p<br>miR-182-5p	miR-183/96/182	[62,220]
FOXQ1	Forkhead box Q1	O	miR-106a-5p	miR-106a/363	[451]
GABBR1	Gamma-amino-butyric acid type B receptor 1	TS	miR-106a-5p<br>miR-20b-5p	miR-106a/363	[452]
			miR-106b-5p	miR-106b/25/93	[452]
			miR-20a-5p<br>miR-17-5p	miR-17/92a-1	[452]
GATA6	GATA Binding Protein 6	O	mi-363-3p	miR-106a/363	[38]
			miR-203a-3p	miR-203a/203b	[453]
GEMIN2	Gem nuclear organelle associated protein 2	O	miR-141-3p	miR-200c/141	[454]
GPC1	Glypican-1	O	miR-96-5p	miR-183/96/182	[262]
GRN	Granulin precursor	O	miR-29b-3p	miR-29b-1/29a	[64]
GSK3B	Glycogen synthase kinase 3 beta	TS	miR-92a-3p	miR-106a/363<br>miR-17/92a-1	[210]
			miR-452-3p<br>miR-224-5p	miR-452/224	[195,221]
HIF1A	Hypoxia inducible factor 1 subunit alpha	O	miR-93-5p	miR-106b/25/93	[81]
			miR-18a-5p	miR-17/92a-1	[230]
HNRNPA1	Heterogeneous nuclear ribonucleoprotein	O	miR-18a-5p	miR-17/92a-1	[455]

	A1				
ING4	Inhibitor of growth family member 4	TS	miR-18a-5p	miR-17/92a-1	[60]
ITGA5	Integrin subunit alpha 5	O	miR-130b-3p	miR-301b/130b	[406]
ITGB1	Integrin subunit beta 1	O	miR-130b-3p	miR-301b/130b	[456]
ITGB8	Integrin subunit beta 8	O	miR-106b-5p	miR-106b/25/93	[53]
KIF14	Kinesin family member 14	O	miR-200c-3p	miR-200c/141	[457]
KLF4	Krüppel like factor 4	TS	miR-92a-3p	miR-106a/363<br>miR-17/92a-1	[210]
			miR-29a-3p	miR-29b-1/29a	[190]
KLK6	Kallikrein related peptidase 6	O	miR-203a-3p	miR-203a/203b	[458]
KRAS	KRAS proto-oncogene, GTPase	O	miR-19a-3p	miR-17/92a-1	[229]
			miR-96-5p	miR-183/96/182	[167]
LGALSL	Galectin like	O	miR-224-5p	miR-452/224	[440]
MAP2K4	Mitogen-activated protein kinase kinase 4	TS	miR-141-3p	miR-200c/141	[380]
MAP3K5	Mitogen-activated protein kinase kinase kinase 5	TS	miR-20a-5p	miR-17/92a-1	[459]
MAP4K4	Mitogen-activated protein kinase kinase kinase kinase 4	O	miR-141-3p	miR-200c/141	[460]
MARK1	Microtubule affinity regulating kinase 1	TS	miR-23a-3p	miR-23a/27a/24-2	[393]
MBD2	Methyl-CpG binding domain protein 2	O	miR-221-5p	miR-221/222	[182]
			miR-224-5p	miR-452/224	[182]
MCU	Mitochondrial calcium uniporter	TS	miR-25-3p	miR-106b/25/93	[334]
MICA	MHC class I polypeptide-related sequence A	TS	miR-20a-5p	miR-17/92a-1	[461]
MMP14	Matrix metallopeptidase 14	O	miR-181a-5p	miR-181a-1/181b-1<br>miR-181a-2/181b-2	[462]
MMP2	Matrix metallopeptidase 2	O	miR-29a-3p	miR-29b-1/29a	[245]
MOAP1	Modulator of apoptosis 1	TS	miR-92a-3p	miR-106a/363<br>miR-17/92a-1	[319]
MTDH	Metadherin	O	miR-182-5p	miR-183/96/182	[366]
MYC	MYC proto-oncogene, bHLH transcription factor	O	miR-93-5p	miR-106b/25/93	[222]
			miR-182-5p	miR-183/96/182	[223]
NCOA3	Nuclear receptor coactivator 3	TS	miR-17-5p	miR-17/92a-1	[33]
NEDD9	Neural precursor cell expressed, developmentally down-regulated 9	O	miR-25-5p	miR-106b/25/93	[463]
			miR-18a-5p	miR-17/92a-1	[439]
			miR-203a-3p	miR-203a/203b	[464]
NF2	Neurofibromin 2	TS	miR-92a-3p	miR-106a/363<br>miR-17/92a-1	[465]
PDCD10	Programmed cell death 10	TS	miR-425-5p	miR-191/425	[466]
PDCD4	Programmed cell death 4	TS	miR-20a-5p	miR-17/92a-1	[65]
			miR-181b-5p	miR-181a-1/181b-1<br>miR-181a-2/181b-2	[356]

			miR-141-3p	miR-200c/141	[76]
PDE4D	Phosphodiesterase 4D	TS	miR-203a-3p	miR-203a/203b	[384]
PDK4	Pyruvate dehydrogenase kinase 4	TS	miR-23a-3p	miR-23a/27a/24-2	[467]
PEAK1	Pseudopodium enriched atypical kinase 1	O	miR-181d-5p	miR-181c/181d	[161]
PHLPP1	PH domain leucine-rich-repeat protein phosphatase 1	TS	miR-224-5p	miR-452/224	[409]
PHLPP2	PH domain leucine-rich-repeat protein phosphatase 2	TS	miR-224-5p	miR-452/224	[409]
PIAS3	Protein inhibitor of activated STAT 3	TS	miR-18a-5p	miR-17/92a-1	[468]
			miR-181b-5p	miR-181a-1/181b-1 miR-181a-2/181b-2	[469]
PLAG1	PLAG1 Zinc Finger	O	miR-181a-5p	miR-181a-1/181b-1 miR-181a-2/181b-2	[470]
PLK3	Polo like kinase 3	TS	miR-106b-5p	miR-106b/25/93	[471]
POU5F1	POU Class 5 Homeobox 1	O	miR-20b-5p	miR-106a/363	[75]
PRICKLE2	Prickle planar cell polarity protein 2 EPM5	O	miR-200c-5p	miR-200c/141	[472]
PRKCZ	Protein kinase C zeta	O	miR-25-5p	miR-106b/25/93	[473]
PRRX1	Paired related homeobox 1	TS	miR-106b-5p	miR-106b/25/93	[234]
PTEN	Phosphatase and tensin homolog	TS	miR-106a-3p	miR-106a/363	[474]
			miR-106b-5p miR-93-5p	miR-106b/25/93	[50,234]
			miR-19a-3p miR-17-5p miR-92a-3p	miR-17/92a-1 miR-106a/363	[151,416, 475]
			miR-181a-5p	miR-181a-1/181b-1 miR-181a-2/181b-2	[355]
			miR-29a-3p	miR-29b-1/29a	[476]
RASSF1	Ras association domain family member 1	O	miR-181b-5p	miR-181a-1/181b-1 miR-181a-2/181b-2	[357]
RB1CC1	RB1 inducible coiled-coil 1	TS	miR-20a-5p	miR-17/92a-1	[433]
RBL2	RB transcriptional corepressor like 2	TS	miR-17-5p	miR-17/92a-1	[150]
RBM4	RNA binding motif protein 4	TS	miR-92a-2-3 p	miR-106a/363 miR-17/92a-1	[320]
RCN2	Reticulocalbin 2	TS	miR-183-5p	miR-183/96/182	[362]
RECK	Reversion inducing cysteine rich protein with kazal motifs	TS	miR-92a-3p	miR-106a/363 miR-17/92a-1	[138]
			miR-96-3p	miR-183/96/182	[477]
RND3	Rho Family GTPase 3	TS	miR-17-5p	miR-17/92a-1	[338]
RNF6	RING finger protein 6	O	miR-203a-3p	miR-203a/203b	[422]
ROBO1	Roundabout guidance receptor 1	O	miR-203a-3p	miR-203a/203b	[478]
RPS15A	Ribosomal protein S15A	O	miR-29a-3p	miR-29b-1/29a	[404]
RXRA	Retinoid X receptor alpha	TS	miR-27a-3p	miR-23a/27a/24-2	[184]

SATB2	SATB homeobox 2	TS	miR-182-5p	miR-183/96/182	[364]
SEMA4C	Semaphorin 4C	O	miR-25-3p	miR-106b/25/93	[61]
SEMA6D	Semaphorin 6D	TS	miR-23a-5p	miR-23a/27a/24-2	[479]
SFRP1	Secreted frizzled related protein 1	TS	miR-27a-3p	miR-23a/27a/24-2	[183]
SFRP2	Secreted frizzled related protein 2	TS	miR-224-5p	miR-452/224	[221]
SGPP1	Sphingosine-1-phosphate phosphatase 1	O	miR-27a-3p	miR-23a/27a/24-2	[480]
SIK1	Salt inducible kinase 1	TS	miR-17-5p	miR-17/92a-1	[152]
SIK2	Salt inducible kinase 2	O	miR-203a-3p	miR-203a/203b	[387]
SIRT1	Sirtuin 1	O	miR-141-3p	miR-200c/141	[78]
			miR-29b-3p	miR-29b-1/29a	[239]
SLAIN2	SLAIN motif family member 2	O	miR-106b-5p	miR-106b/25/93	[74]
SLC4A4	Solute carrier family 4 member 4	O	miR-224-5p	miR-452/224	[440]
SMAD2	SMAD family member 2	O	miR-27a-3p	miR-23a/27a/24-2	[480]
SMAD3	SMAD family member 3	TS	miR-29b-1-5p	miR-29b-1/29a	[481]
SMAD4	SMAD family member 4	TS	miR-18a-5p<br>miR-19b-3p<br>miR-20a-5p	miR-17/92a-1<br>miR-106a/363	[155,227,228,415]
			miR-224-5p	miR-452/224	[198,482]
SMAD7	SMAD family member 7	TS	miR-92a-3p	miR-106a/363<br>miR-17/92a-1	[483]
		O	miR-25-3p<br>miR-93-5p	miR-106b/25/93	[222,336]
SOX2	SRY-box transcription factor 2	O	miR-200c-3p	miR-200c/141	[418]
SOX4	SRY-box transcription factor 4	O	miR-363-3p	miR-106a/363	[327]
SOX9	SRY-box transcription factor 9	O	miR-18b-5p	miR-106a/363	[57]
SPHK2	Sphingosine kinase 2	O	miR-363-3p	miR-106a/363	[144]
SRCIN1	SRC kinase signalling inhibitor 1	TS	miR-181a-5p	miR-181a-1/181b-1<br>miR-181a-2/181b-2	[484]
ST6GALNAC2	ST6 N-acetylgalactosaminide alpha-2,6-sialyltransferase 2	TS	miR-182-5p	miR-183/96/182	[365]
STAT1	Signal transducer and activator of transcription 1	TS	miR-181a-5p	miR-181a-1/181b-1<br>miR-181a-2/181b-2	[485]
TBPL1	TATA box-binding protein-like protein 1	O	miR-18a-5p	miR-17/92a-1	[340]
TCF4	Transcription factor 4	O	miR-181a-5p	miR-181a-1/181b-1<br>miR-181a-2/181b-2	[56]
			miR-203a-3p	miR-203a/203b	[225]
TF	Tissue factor	O	miR-19a-3p	miR-17/92a-1	[486]
TGFBR2	Transforming growth factor beta receptor 2	TS	miR-106a-5p	miR-106a/363	[209,411]
		O	miR-17-5p<br>miR-20-5p	miR-17/92a-1	[228,230]

			miR-19a-3p		
TGM2	Transglutaminase 2	TS	miR-19a-3p	miR-17/92a-1	[487]
THBS1	Thrombospondin 1	TS	miR-182-5p	miR-183/96/182	[237]
			miR-19a-3p	miR-17/92a-1	[236]
THBS2	Thrombospondin 2	O	miR-203a-3p	miR-203a/203b	[238]
TIA1	TIA1 cytotoxic granule associated RNA binding protein	TS	miR-19a-3p	miR-17/92a-1	[488]
TIAM1	TIAM Rac1 associated GEF 1	O	miR-29b-3p	miR-29b-1/29a	[401]
TIMP3	TIMP metallopeptidase inhibitor 3	TS	miR-191-5p	miR-191/425	[417]
TLE5	TLE family member 5, transcriptional modulator	O	miR-203a-3p	miR-203a/203b	[67]
TNFAIP3	TNF alpha induced protein 3	TS	miR-19a-3p	miR-17/92a-1	[343]
		O	miR-29a-3p	miR-29b-1/29a	[69]
TP53	Tumor protein P53	TS	miR-27a-3p	miR-23a/27a/24-2	[396]
TP53INP1	Tumor protein p53 inducible nuclear protein 1	TS	miR-96-5p	miR-183/96/182	[220]
TPM1	Tropomyosin 1	TS	miR-96-5p	miR-183/96/182	[363]
TRAF5	TNF receptor associated factor 5	O	miR-141-3p	miR-200c/141	[179]
TRIM8	Tripartite motif containing 8	TS	miR-106b-5p	miR-106b/25/93	[489]
		TS	miR-17-5p	miR-17/92a-1	[489]
TUSC3	Tumor suppressor candidate 3	O	miR-181b-5p	miR-181a-1/181b-1<br>miR-181a-2/181b-2	[41]
TYMS	Thymidylate synthetase	O	miR-203a-3p	miR-203a/203b	[490]
USP3	Ubiquitin specific peptidase 3	TS	miR-224-5p	miR-452/224	[482]
UVRAG	UV radiation resistance associated	TS	miR-183-5p	miR-183/96/182	[361]
VANGL	VANGL planar cell polarity protein 1	O	miR-27a-3p	miR-23a/27a/24-2	[491]
VEGFA	Vascular endothelial growth factor A	O	miR-20a-5p	miR-17/92a-1	[230]
VHL	von Hippel Lindau tumour suppressor	TS	miR-24-3p	miR-23a/27a/24-2	[394]
VIM	Vimentin	O	miR-17-5p	miR-17/92a-1	[413]
VLDLR	Very low-density lipoprotein receptor	TS	miR-200c-3p	miR-200c/141	[372]
WIF1	WNT inhibitory factor 1	TS	miR-181a-5p	miR-181a-1/181b-1<br>miR-181a-2/181b-2	[160]
ZEB1	Zinc finger E-box binding homeobox 1	O	miR-200c-3p<br>miR-141-3p	miR-200c/141	[36,47]

Function of genes in human CRC cell lines was described according referred study, tumour suppressor function was abbreviated as TS and genes with oncogenic function were abbreviated as O.

Supplementary material S5: List of abbreviated lncRNA and circRNA genes

ACVRL1	Activin A receptor like type 1
AGER	Advanced glycosylation end-product specific receptor
BANCR	BRAF-activated non-protein coding RNA
CA3-AS1	CA3 antisense RNA 1
CCAT1	Colon cancer associated transcript 1
CDK6	Cyclin dependent kinase 6
CRNDE	Colorectal neoplasia differentially expressed
DUSP2	Dual specificity phosphatase 2
FARSA-AS1	FARSA antisense RNA 1
FBXL19-AS1	FBXL19 antisense RNA 1
FBXW7	F-box and WD repeat domain containing 7
FENDRR	FOXF1 adjacent non-coding developmental regulatory RNA
FOXD2-AS1	FOXD2 adjacent opposite strand RNA 1
GAS5	Growth arrest specific 5
H19	H19 imprinted maternally expressed transcript
HAND2-AS1	HAND2 antisense RNA 1
HOTAIR	HOX transcript antisense RNA
HOTAIRM1	HOXA transcript antisense RNA, myeloid-specific 1
LIFR-AS1	LIFR antisense RNA 1
LINC00858	Long intergenic non-protein coding RNA 858
LINC01567	Long intergenic non-protein coding RNA 1567
LINC02595	Long intergenic non-protein coding RNA 2595
LINC-PINT	Long intergenic non-protein coding RNA, p53 induced transcript
lncRNA ATB	Long noncoding RNA Activated by TGF-beta
MALAT1	Metastasis associated lung adenocarcinoma transcript 1
MEG3	Maternally expressed 3
NORAD	Non-coding RNA activated by DNA damage
POLR2J4	RNA polymerase II subunit J4, pseudogene
SMARCA5	SWI/SNF related, matrix associated, actin dependent regulator of chromatin, subfamily a, member 5
SNHG15	Small nucleolar RNA host gene 15
UCA1	Urothelial cancer associated 1
XIRP2-AS1	XIRP2 antisense RNA 1
XIST	X inactive specific transcript
ZEB1-AS1	ZEB1 antisense RNA 1

Supplementary material S6: List of abbreviated target genes of selected miRNA clusters

ABCA1	ATP binding cassette subfamily A member 1
ABCF1	ATP-binding cassette, subfamily F, member 1
ADAM9	ADAM metallopeptidase domain 9
AFDN	Afadin, Adherens junction formation factor
AGER	Advanced glycosylation end-product specific receptor
AKAP12	A kinase anchor protein 12
AKT2	AKT serine/threonine kinase 2
AMER1	APC membrane recruitment protein 1
ANGPTL8	Angiopoietin like 8
APAF1	Apoptotic peptidase activating factor 1
AQP8	Aquaporin 8
ARID4	AT-rich interaction domain 4B
ATG12	Autophagy related 12
ATG16L1	Autophagy Related 16 Like 1
ATG5	Autophagy related 5
ATG7	Autophagy related 7
ATM	ATM serine/threonine kinase
ATXN3	Ataxin 3
BCL2L1	BCL2 like 1
BCL2L11	BCL2 like 11
BCL9L	BCL9 like
BID	BH3 interacting domain death agonist
BMI1	BMI1 proto-oncogene, polycomb ring finger
BNIP2	BCL2 interacting protein 2
BTG1	BTG anti-proliferation factor 1
BTG3	BTG anti-proliferation factor 3
CEBPB	CCAAT enhancer binding protein beta
CALR	Calreticulin
CCND1	Cyclin D1
CCND2	Cyclin D2
CD274	CD274 molecule
CDC42	Cell division cycle 42
CDH1	Cadherin 1
CDK19	Cyclin dependent kinase 19
CDK2	Cyclin Dependent Kinase 2
CDKN1A	Cyclin dependent kinase inhibitor 1A

CDKN2B	Cyclin dependent kinase inhibitor 2B
CDS2	CDP-diacylglycerol synthase 2
CLU	Clusterin
CPEB4	Cytoplasmic polyadenylation element-binding 4
CREB1	cAMP responsive element binding protein 1
CRY2	Cryptochrome circadian regulator 2
CSE1L	Chromosomal segregation 1 like
CTNNB1	Catenin Beta 1
CTNND1	Catenin delta 1
CTSA	Cathepsin A
CXCL8	C-X-C motif chemokine ligand 8
CYP7B1	Cytochrome P450, family 7, subfamily B, member 1
DAB2IP	DAB2 interacting protein
DHFR	Dihydrofolate reductase
DKK3	Dickkopf WNT signalling pathway inhibitor 3
DLC1	DLC1 Rho GTPase activating protein
DND1	DND microRNA-mediated repression inhibitor 1
DNMT3B	DNA methyltransferase 3 beta
DUSP2	Dual-specificity phosphatase 2
E2F5	E2F transcription factor 5
EGFR	Epidermal growth factor receptor
EIF5A2	Eukaryotic initiation factor 5A2
ETS1	ETS proto-oncogene 1, transcription Factor
EZH2	Enhancer of zeste 2 polycomb repressive complex 2 subunit
F2RL3	F2R like thrombin or trypsin receptor 3
FAM172A	Family with sequence similarity 172 member A
FBXL3	F-box and leucine rich repeat protein 3
FBXW7	F-Box and WD repeat domain containing 7
FLT1	Fms related receptor tyrosine kinase 1
FOXA1	Forkhead box A1
FOXF2	Forkhead box F2
FOXJ2	Forkhead Box J2
FOXO1	Forkhead box protein O1
FOXO3a	Forkhead box protein O3a
FOXQ1	Forkhead box Q1
GABBR1	Gamma-amino-butyric acid type B receptor 1
GATA6	GATA Binding Protein 6
GEMIN2	Gem nuclear organelle associated protein 2

GPC1	Glypican-1
GRN	Granulin precursor
GSK3B	Glycogen synthase kinase 3 beta
HIF1A	Hypoxia inducible factor 1 subunit alpha
HNRNPA1	Heterogeneous nuclear ribonucleoprotein A1
ING4	Inhibitor of growth family member 4
ITGA5	Integrin subunit alpha 5
ITGB1	Integrin subunit beta 1
ITGB8	Integrin subunit beta 8
KIF14	Kinesin family member 14
KLF4	Krüppel like factor 4
KLK6	Kallikrein related peptidase 6
KRAS	KRAS proto-oncogene, GTPase
LGALSL	Galectin like
MAP2K4	Mitogen-activated protein kinase kinase 4
MAP3K5	Mitogen-activated protein kinase kinase kinase 5
MAP4K4	Mitogen-activated protein kinase kinase kinase kinase 4
MARK1	Microtubule affinity regulating kinase 1
MBD2	Methyl-CpG binding domain protein 2
MCU	Mitochondrial calcium uniporter
MICA	MHC class I polypeptide-related sequence A
MMP14	Matrix metallopeptidase 14
MMP2	Matrix metallopeptidase 2
MOAP1	Modulator of apoptosis 1
MTDH	Metadherin
MYC	MYC proto-oncogene, bHLH transcription factor
NCOA3	Nuclear receptor coactivator 3
NEDD9	Neural precursor cell expressed, developmentally down-regulated 9
NF2	Neurofibromin 2
PDCD10	Programmed cell death 10
PDCD4	Programmed cell death 4
PDE4D	Phosphodiesterase 4D
PDK4	Pyruvate dehydrogenase kinase 4
PEAK1	Pseudopodium enriched atypical kinase 1
PHLPP1	PH domain leucine-rich-repeat protein phosphatase 1
PHLPP2	PH domain leucine-rich-repeat protein phosphatase 2
PIAS3	Protein inhibitor of activated STAT 3
PLAG1	PLAG1 Zinc Finger

PLK3	Polo like kinase 3
POU5F1	POU Class 5 Homeobox 1
PRICKLE2	Prickle planar cell polarity protein 2 EPM5
PRKCZ	Protein kinase C zeta
PRRX1	Paired related homeobox 1
PTEN	Phosphatase and tensin homolog
RASSF1	Ras association domain family member 1
RB1CC1	RB1 inducible coiled-coil 1
RBL2	RB transcriptional corepressor like 2
RBM4	RNA binding motif protein 4
RCN2	Reticulocalbin 2
RECK	Reversion inducing cysteine rich protein with kazal motifs
RND3	Rho Family GTPase 3
RNF6	RING finger protein 6
ROBO1	Roundabout guidance receptor 1
RPS15A	Ribosomal protein S15A
RXRA	Retinoid X receptor alpha
SATB2	SATB homeobox 2
SEMA4C	Semaphorin 4C
SEMA6D	Semaphorin 6D
SFRP1	Secreted frizzled related protein 1
SFRP2	Secreted frizzled related protein 2
SGPP1	Sphingosine-1-phosphate phosphatase 1
SIK1	Salt inducible kinase 1
SIK2	Salt inducible kinase 2
SIRT1	Sirtuin 1
SLAIN2	SLAIN motif family member 2
SLC4A4	Solute carrier family 4 member 4
SMAD2	SMAD family member 2
SMAD3	SMAD family member 3
SMAD4	SMAD family member 4
SMAD7	SMAD family member 7
SOX2	SRY-box transcription factor 2
SOX4	SRY-box transcription factor 4
SOX9	SRY-box transcription factor 9
SPHK2	Sphingosine kinase 2
SRCIN1	SRC kinase signalling inhibitor 1
ST6GALNAC2	ST6 N-acetylgalactosaminide alpha-2,6-sialyltransferase 2

STAT1	Signal transducer and activator of transcription 1
TBPL1	TATA box-binding protein-like protein 1
TCF4	Transcription factor 4
TF	Tissue factor
TGFBR2	Transforming growth factor beta receptor 2
TGM2	Transglutaminase 2
THBS1	Thrombospondin 1
THBS2	Thrombospondin 2
TIA1	TIA1 cytotoxic granule associated RNA binding protein
TIAM1	TIAM Rac1 associated GEF 1
TIMP3	TIMP metallopeptidase inhibitor 3
TLE5	TLE family member 5, transcriptional modulator
TNFAIP3	TNF alpha induced protein 3
TP53	Tumor protein P53
TP53INP1	Tumor protein p53 inducible nuclear protein 1
TPM1	Tropomyosin 1
TRAF5	TNF receptor associated factor 5
TRIM8	Tripartite motif containing 8
TUSC3	Tumor suppressor candidate 3
TYMS	Thymidylate synthetase
USP3	Ubiquitin specific peptidase 3
UVRAG	UV radiation resistance associated
VANGL	VANGL planar cell polarity protein 1
VEGFA	Vascular endothelial growth factor A
VHL	von Hippel Lindau tumour suppressor
VIM	Vimentin
VLDLR	Very low-density lipoprotein receptor
WIF1	WNT inhibitory factor 1
ZEB1	Zinc finger E-box binding homeobox 1
ZNF217	Zinc finger protein 217

Supplementary material S8: Refferences cited in Supplemental materials, which were not cited in the main text

270. Motoyama, K.; Inoue, H.; Takatsuno, Y.; Tanaka, F.; Mimori, K.; Uetake, H.; Sugihara, K.; Mori, M.: Over- and under-expressed microRNAs in human colorectal cancer. *Int J Oncol*. **2009,** *34*, 1069–1075, doi: 10.3892/ijo_00000233.
271. Arndt, G.M.; Dossey, L.; Cullen, L.M.; Lai, A.; Druker, R.; Eisbacher, M.; Zhang, C.; Tran, N.; Fan, H.; Retzlaff, K.; et al. Characterization of global microRNA expression reveals oncogenic potential of miR-145 in metastatic colorectal cancer. *BMC Cancer* **2009**, *9*, 1–17, doi:10.1186/1471-2407-9-374.
272. Wang, Y.X.; Zhang, X.Y.; Zhang, B.F.; Yang, C.Q.; Chen, X.M.; Gao, H.J. Initial study of microRNA expression profiles of colonic cancer without lymph node metastasis. *J. Dig. Dis.* **2010**, *11*, 50–54, doi:10.1111/j.1751-2980.2009.00413.x.
273. Huang, Z.; Huang, S.; Wang, Q.; Liang, L.; Ni, S.; Wang, L.; Sheng, W.; He, X.; Du, X. MicroRNA-95 Promotes Cell Proliferation and Targets Sorting Nexin 1 in Human Colorectal Carcinoma. *Cancer Res.* **2011**, *71*, 2582–2589, doi:10.1158/0008-5472.can-10-3032.
274. Fu, J.; Tang, W.; Du, P.; Wang, G.; Chen, W.; Li, J.; Zhu, Y.; Gao, J.; Cui, L. Identifying MicroRNA-mRNA regulatory network in colorectal cancer by a combination of expression profile and bioinformatics analysis. *BMC Syst. Biol.* **2012**, *6*, 68, doi:10.1186/1752-0509-6-68.
275. Hamfjord, J.; Stangeland, A.M.; Hughes, T.; Skrede, M.L.; Tveit, K.M.; Ikdahl, T.; Kure, E.H. Differential Expression of miRNAs in Colorectal Cancer: Comparison of Paired Tumor Tissue and Adjacent Normal Mucosa Using High-Throughput Sequencing. *PLoS ONE* **2012**, *7*, e34150, doi:10.1371/journal.pone.0034150.
276. Chen, W.C.; Lin, M.S.; Ye, Y.L.; Gao, H.J.; Song, Z.Y.; Shen, X.Y. microRNA expression pattern and its alteration following celecoxib intervention in human colorectal cancer. *Exp. Ther. Med.* **2012**, *3*, 1039–1048, doi:10.3892/etm.2012.531.
277. Wu, W.; Li, X.; Zhang, G.; Luo, F.; Ruan, J.; Huang, D.; Feng, D.; Xiao, D.; Zeng, Z.; Chen, X. Identification of aberrantly expressed miRNAs in rectal cancer. *Oncol. Rep.* **2012**, *28*, 77–84, doi:10.3892/or.2012.1769.
278. Mosakhani, N.; Sarhadi, V.K.; Borze, I.; Karjalainen-Lindsberg, M.-L.; Sundström, J.; Ristamäki, R.; Österlund, P.; Knuutila, S. MicroRNA profiling differentiates colorectal cancer according to KRAS status. *Genes Chromosom. Cancer* **2011**, *51*, 1–9, doi:10.1002/gcc.20925.
279. Nishida, N.; Nagahara, M.; Sato, T.; Mimori, K.; Sudo, T.; Tanaka, F.; Shibata, K.; Ishii, H.; Sugihara, K.; Doki, Y.; et al. Microarray Analysis of Colorectal Cancer Stromal Tissue Reveals Upregulation of Two Oncogenic miRNA Clusters. *Clin. Cancer Res.* **2012**, *18*, 3054–3070, doi:10.1158/1078-0432.ccr-11-1078.
280. Chen, W.-S.; Chen, T.-W.; Yang, T.-H.; Hu, L.-Y.; Pan, H.-W.; Leung, C.-M.; Li, S.-C.; Ho, M.-R.; Shu, C.-W.; Liu, P.-F.; et al. Co-modulated behavior and effects of differentially expressed miRNA in colorectal cancer. *BMC Genom.* **2013**, *14*, S12, doi:10.1186/1471-2164-14-s5-s12.
281. Ak, S.; Tunca, B.; Tezcan, G.; Cecener, G.; Egeli, U.; Yilmazlar, T.; Ozturk, E.; Yerci, O. MicroRNA expression patterns of tumors in early-onset colorectal cancer patients. *J. Surg. Res.* **2014**, *191*, 113–122, doi:10.1016/j.jss.2014.03.057.
282. Li, E.; Ji, P.; Ouyang, N.; Zhang, Y.; Wang, X.Y.; Rubin, D.C.; Davidson, N.O.; Bergamaschi, R.; Shroyer, K.R.; Burke, S.; et al. Differential expression of miRNAs in colon cancer between African and Caucasian Americans: Implications for cancer racial health disparities. *Int. J. Oncol.* **2014**, *45*, 587–594, doi:10.3892/ijo.2014.2469.
283. Fukushima, Y.; Iinuma, H.; Tsukamoto, M.; Matsuda, K.; Hashiguchi, Y. Clinical significance of microRNA-21 as a biomarker in each Dukes' stage of colorectal cancer. *Oncol. Rep.* **2014**, *33*, 573–582, doi:10.3892/or.2014.3614.
284. Mo, J.-S.; Alam, K.J.; Kang, I.-H.; Park, W.C.; Seo, G.-S.; Choi, S.-C.; Kim, H.-S.; Moon, H.-B.; Yun, K.-J.; Chae, S.-C. MicroRNA 196B regulates FAS-mediated apoptosis in colorectal cancer cells. *Oncotarget* **2015**, *6*, 2843–2855, doi:10.18632/oncotarget.3066.
285. Peng, J.; Xie, Z.; Cheng, L.; Zhang, Y.; Chen, J.; Yu, H.; Li, Z.; Kang, H. Paired design study by real-time PCR: miR-378* and miR-145 are potent early diagnostic biomarkers of human colorectal cancer. *BMC Cancer* **2015**, *15*, 158, doi:10.1186/s12885-015-1123-2.
286. Tanoglu, A.; Balta, A.Z.; Berber, U.; Ozdemir, Y.; Emirzeoglu, L.; Sayilir, A.; Sucullu, I. microRNA Expression Profile in Patients with Stage II Colorectal Cancer: A Turkish Referral Center Study. *Asian Pac. J. Cancer Prev.* **2015**, *16*, 1851–1855, doi:10.7314/apjcp.2015.16.5.1851.
287. Wu, X.; Li, S.; Xu, X.; Wu, S.; Chen, R.; Jiang, Q.; Li, Y.; Xu, Y. The potential value of miR-1 and miR-374b as biomarkers for colorectal cancer. *Int. J. Clin. Exp. Pathol.* **2015**, *8*, 2840–2851.
288. Neerincx, M.; Sie, D.L.S.; Van De Wiel, M.A.; Van Grieken, N.C.T.; Burggraaf, J.D.; Dekker, H.; Eijk, P.P.; Ylstra, B.; Verhoef, C.; Meijer, G.A.; et al. MiR expression profiles of paired primary colorectal cancer and metastases by next-generation sequencing. *Oncogene* **2015**, *4*, e170, doi:10.1038/oncsis.2015.29.
289. Meng, W.-J.; Yang, L.; Ma, Q.; Zhang, H.; Adell, G.; Arbman, G.; Wang, Z.-Q.; Li, Y.; Zhou, Z.-G.; Sun, X.-F. MicroRNA Expression Profile Reveals miR-17-92 and miR-143-145 Cluster in Synchronous Colorectal Cancer. *Med.* **2015**, *94*, e1297, doi:10.1097/md.0000000000001297.
290. Kara, M.; Yumrutas, O.; Ozcan, O.; Celik, O.I.; Bozgeyik, E.; Bozgeyik, I.; Tasdemir, S. Differential expressions of cancer-associated genes and their regulatory miRNAs in colorectal carcinoma. *Gene* **2015**, *567*, 81–86, doi:10.1016/j.gene.2015.04.065.
291. Ozcan, O.; Kara, M.; Yumrutas, O.; Bozgeyik, E.; Bozgeyik, I.; Celik, O.I. MTUS1 and its targeting miRNAs in colorectal carcinoma: Significant associations. *Tumor Biol.* **2016**, *37*, 6637–6645, doi:10.1007/s13277-015-4550-4.
292. Pelossof, R.; Chow, O.S.; Fairchild, L.; Smith, J.J.; Setty, M.; Chen, C.-T.; Chen, Z.; Egawa, F.; Avila, K.; Leslie, C.S.; et al. Integrated genomic profiling identifies microRNA-92a regulation of IQGAP2 in locally advanced rectal cancer. *Genes, Chromosom. Cancer* **2016**, *55*, 311–321, doi:10.1002/gcc.22329.

293. Sun, G.; Cheng, Y.-W.; Lai, L.; Huang, T.-C.; Wang, J.; Wu, X.; Wang, Y.; Huang, Y.; Wang, J.; Zhang, K.; et al. Signature miRNAs in colorectal cancers were revealed using a bias reduction small RNA deep sequencing protocol. *Oncotarget* **2015**, *7*, 3857–3872, doi:10.18632/oncotarget.6460.
294. Zekri, A.-R.N.; Youssef, A.S.E.-D.; Lotfy, M.M.; Gabr, R.; Ahmed, O.S.; Nassar, A.; Hussein, N.; Omran, D.; Medhat, E.; Eid, S.; et al. Circulating Serum miRNAs as Diagnostic Markers for Colorectal Cancer. *PLoS ONE* **2016**, *11*, e0154130, doi:10.1371/journal.pone.0154130.
295. Li, Y.; Chen, M.; Liu, J.; Li, L.; Yang, X.; Zhao, J.; Wu, M.; Ye, M. Upregulation of MicroRNA 18b Contributes to the Development of Colorectal Cancer by Inhibiting CDKN2B. *Mol. Cell. Biol.* **2017**, *37*, e00391-17, doi:10.1128/mcb.00391-17.
296. Nagy, Z.B.; Wichmann, B.; Kalmár, A.; Galamb, O.; Barták, B.K.; Spisák, S.; Tulassay, Z.; Molnár, B. Colorectal adenoma and carcinoma specific miRNA profiles in biopsy and their expression in plasma specimens. *Clin. Epigenetics* **2017**, *9*, 1–14, doi:10.1186/s13148-016-0305-3.
297. Wu, F.; Yuan, G.; Chen, J.; Wang, C. Network analysis based on TCGA reveals hub genes in colon cancer. *Współczesna Onkologia* **2017**, *21*, 136–144, doi:10.5114/wo.2017.68622.
298. Du, B.; Wu, D.; Yang, X.; Wang, T.; Shi, X.; Lv, Y.; Zhou, Z.; Liu, Q.; Zhang, W. The expression and significance of microRNA in different stages of colorectal cancer. *Med.* **2018**, *97*, e9635, doi:10.1097/md.0000000000009635.
299. Pellatt, A.J.; Mullany, L.E.; Herrick, J.S.; Sakoda, L.C.; Wolff, R.K.; Samowitz, W.S.; Slattery, M.L. The TGFβ-signaling pathway and colorectal cancer: Associations between dysregulated genes and miRNAs. *J. Transl. Med.* **2018**, *16*, 191, doi:10.1186/s12967-018-1566-8.
300. Zhang, Y.; Guo, L.; Li, Y.; Feng, G.-H.; Teng, F.; Li, W.; Zhou, Q. MicroRNA-494 promotes cancer progression and targets adenomatous polyposis coli in colorectal cancer. *Mol. Cancer* **2018**, *17*, 1–11, doi:10.1186/s12943-017-0753-1.
301. Brînzan, C.; Aşchie, M.; Matei, E.; Mitroi, A.; Cozaru, G. Molecular expression profiles of selected microRNAs in colorectal adenocarcinoma in patients from south-eastern part of Romania. *Medicine* **2019**, *98*, e18122, doi:10.1097/md.0000000000018122.
302. Li, J.; Zhong, Y.; Cai, S.; Zhou, P.; Yao, L. MicroRNA expression profiling in the colorectal normal-adenoma-carcinoma transition. *Oncol. Lett.* **2019**, *18*, 2013–2018, doi:10.3892/ol.2019.10464.
303. Zhang, H.; Zhu, M.; Shan, X.; Zhou, X.; Wang, T.; Zhang, J.; Tao, J.; Cheng, W.; Chen, G.; Li, J.; et al. A panel of seven-miRNA signature in plasma as potential biomarker for colorectal cancer diagnosis. *Gene* **2019**, *687*, 246–254, doi:10.1016/j.gene.2018.11.055.
304. Gungormez, C.; Aktas, H.G.; Dilsiz, N.; Borazan, E. Novel miRNAs as potential biomarkers in stage II colon cancer: Microarray analysis. *Mol. Biol. Rep.* **2019**, *46*, 4175–4183, doi:10.1007/s11033-019-04868-7.
305. Hao, S.; Huo, S.; Du, Z.; Yang, Q.; Ren, M.; Liu, S.; Liu, T.; Zhang, G. MicroRNA-related transcription factor regulatory networks in human colorectal cancer. *Med.* **2019**, *98*, e15158, doi:10.1097/md.0000000000015158.
306. Wei, L.; Chen, Z.; Cheng, N.; Li, X.; Chen, J.; Wu, D.; Dong, M.; Wu, X. MicroRNA-126 Inhibit Viability of Colorectal Cancer Cell by Repressing mTOR Induced Apoptosis and Autophagy. *Onco. Targ. Ther.* **2020**, *13*, 2459–2468, doi:10.2147/OTT.S238348.
307. Milanesi, E.; Dobre, M.; Bucuroiu, A.I.; Herlea, V.; Manuc, T.E.; Salvi, A.; De Petro, G.; Manuc, M.; Becheanu, G. miRNAs-Based Molecular Signature for KRAS Mutated and Wild Type Colorectal Cancer: An Explorative Study. *J. Immunol. Res.* **2020**, *2020*, 1–9, doi:10.1155/2020/4927120.
308. Zhou, F.; Tang, D.; Xu, Y.; He, H.; Wu, Y.; Lin, L.; Dong, J.; Tan, W.; Dai, Y. Identification of microRNAs and their Endonucleolytic Cleavaged target mRNAs in colorectal cancer. *BMC Cancer* **2020**, *20*, 242–15, doi:10.1186/s12885-020-06717-4.
309. Kral, J.; Korenkova, V.; Novosadova, V.; Langerova, L.; Schneiderova, M.; Liska, V.; Levy, M.; Veskrnova, V.; Spicak, J.; Opattova, A.; et al. Expression profile of miR-17/92 cluster is predictive of treatment response in rectal cancer. *Carcinogene* **2018**, *39*, 1359–1367, doi:10.1093/carcin/bgy100.
310. Zhu, G.-F.; Xu, Y.-W.; Li, J.; Niu, H.-L.; Ma, W.-X.; Xu, J.; Zhou, P.-R.; Liu, X.; Ye, D.-L.; Liu, X.-R.; et al. Mir20a/106a-WTX axis regulates RhoGDIa/CDC42 signaling and colon cancer progression. *Nat. Commun.* **2019**, *10*, 1–14, doi:10.1038/s41467-018-07998-x.
311. Qin, Y.; Chen, X.; Liu, Z.; Tian, X.; Huo, Z. miR-106a Reduces 5-Fluorouracil (5-FU) Sensitivity of Colorectal Cancer by Targeting Dual-Specificity Phosphatases 2 (DUSP2). *Med. Sci. Monit.* **2018**, *24*, 4944–4951, doi:10.12659/msm.910016.
312. Xusheng, Z.; Wang, G.; Zhang, L.; Zhai, C.; Zhang, J.; Zhao, X.; Jiang, X.; Zhao, Z. Biological effects and clinical characteristics of microRNA-106a in human colorectal cancer. *Oncol. Lett.* **2017**, *14*, 830–836, doi:10.3892/ol.2017.6179.
313. Huang, G.; Ma, J.; Zhang, L. Integrin Subunit Alpha 5 (ITGA5) Gene Circular RNA Sponges microRNA-107 in Colorectal Carcinoma Cells and Tissues and Regulates the Expression of the Forkhead Box J3 (FOXJ3) Gene. *Med. Sci. Monit.* **2020**, *26*, doi:10.12659/msm.920623.
314. Jo, P.; Azizian, A.; Salendo, J.; Kramer, F.; Bernhardt, M.; Wolff, H.A.; Gruber, J.; Grade, M.; Beißbarth, T.; Ghadimi, B.M.; et al. Changes of Microrna Levels in Plasma of Patients with Rectal Cancer during Chemoradiotherapy. *Int. J. Mol. Sci.* **2017**, *18*, 1140, doi:10.3390/ijms18061140.
315. Yamaguchi, T.; Iijima, T.; Wakaume, R.; Takahashi, K.; Matsumoto, H.; Nakano, D.; Nakayama, Y.; Mori, T.; Horiguchi, S.; Miyaki, M. Underexpression of miR-126 and miR-20b in Hereditary and Nonhereditary Colorectal Tumors. *Oncology* **2014**, *87*, 58–66, doi:10.1159/000363303.
316. Li, J.; Feng, Y.; Heng, D.; Chen, R.; Wang, Y.; Xu, Z.; Zhang, D.; Zhang, C.; Zhang, Y.; Ji, D.; et al. Circulating non-coding RNA cluster predicted the tumorigenesis and development of colorectal carcinoma. *Aging* **2020**, *12*, 23047–23066, doi:10.18632/aging.104055.
317. Zhang, J.; Wang, Z.; Han, X.; Jiang, L.; Ge, R.; Wang, X.; Li, J. Up-regulation of microRNA-19b is associated with metastasis and predicts poor prognosis in patients with colorectal cancer. *Int. J. Clin. Exp. Pathol* **2018**, *11*, 3952–3960.

318. Jiang, H.; Wang, P.; Wang, Q.; Wang, B.; Mu, J.; Zhuang, X.; Zhang, L.; Yan, J.; Miller, D.; Zhang, H.-G. Quantitatively Controlling Expression of miR-17~92 Determines Colon Tumor Progression in a Mouse Tumor Model. *Am. J. Pathol.* **2014**, *184*, 1355–1368, doi:10.1016/j.ajpath.2014.01.037.
319. Hu, J.L.; Wang, W.; Lan, X.L.; Zeng, Z.C.; Liang, Y.S.; Yan, Y.R.; Song, F.Y.; Wang, F.F.; Zhu, X.H.; Liao, W.J.; et al. CAFs secreted exosomes promote metastasis and chemotherapy resistance by enhancing cell stemness and epithelial-mesenchymal transition in colorectal cancer. *Mol. Cancer* **2019**, *18*, 1–15, doi:10.1186/s12943-019-1019-x.
320. Liang, Y.-C.; Lin, W.-C.; Lin, Y.-J.; Lin, J.-C. The impact of RNA binding motif protein 4-regulated splicing cascade on the progression and metabolism of colorectal cancer cells. *Oncotarget* **2015**, *6*, 38046–38060, doi:10.18632/oncotarget.5710.
321. Jepsen, R.K.; Novotny, G.; Klarskov, L.L.; Christensen, I.J.; Riis, L.B.; Høgdall, E. Intra-tumor heterogeneity of microRNA-92a, microRNA-375 and microRNA-424 in colorectal cancer. *Exp. Mol. Pathol.* **2016**, *100*, 125–131, doi:10.1016/j.yexmp.2015.12.004.
322. Eylem, C.C.; Yilmaz, M.; Derkus, B.; Nemutlu, E.; Camci, C.B.; Yilmaz, E.; Turkoglu, M.A.; Aytac, B.; Ozyurt, N.; Emregul, E. Untargeted multi-omic analysis of colorectal cancer-specific exosomes reveals joint pathways of colorectal cancer in both clinical samples and cell culture. *Cancer Lett.* **2020**, *469*, 186–194, doi:10.1016/j.canlet.2019.10.038.
323. Vega, A.B.; Pericay, C.; Moya, I.; Ferrer, A.; Dotor, E.; Pisa, A.; Casalots, A.; Serra-Aracil, X.; Oliva, J.-C.; Ruiz, A.; et al. microRNA expression profile in stage III colorectal cancer: Circulating miR-18a and miR-29a as promising biomarkers. *Oncol. Rep.* **2013**, *30*, 320–326, doi:10.3892/or.2013.2475.
324. Wang, S.; Xiang, J.; Li, Z.; Lu, S.; Hu, J.; Gao, X.; Yu, L.; Wang, L.; Wang, J.; Wu, Y.; et al. A plasma microRNA panel for early detection of colorectal cancer. *Int. J. Cancer* **2013**, *136*, 152–161, doi:10.1002/ijc.28136.
325. Luo, X.; Stock, C.; Burwinkel, B.; Brenner, H. Identification and Evaluation of Plasma MicroRNAs for Early Detection of Colorectal Cancer. *PLoS ONE* **2013**, *8*, e62880, doi:10.1371/journal.pone.0062880.
326. Fu, F.; Jiang, W.; Zhou, L.; Chen, Z. Circulating Exosomal miR-17-5p and miR-92a-3p Predict Pathologic Stage and Grade of Colorectal Cancer. *Transl. Oncol.* **2018**, *11*, 221–232, doi:10.1016/j.tranon.2017.12.012.
327. Hu, F.; Min, J.; Cao, X.; Liu, L.; Ge, Z.; Hu, J.; Li, X. MiR-363-3p inhibits the epithelial-to-mesenchymal transition and suppresses metastasis in colorectal cancer by targeting Sox4. *Biochem. Biophys. Res. Commun.* **2016**, *474*, 35–42, doi:10.1016/j.bbrc.2016.04.055.
328. Li, G. Expression of RUNX3 gene and miR-363 in colorectal cancer and the relationship with clinicopathological features. *Oncol. Lett.* **2019**, *18*, 2278–2285, doi:10.3892/ol.2019.10566.
329. Zhang, G.-J.; Li, J.-S.; Zhou, H.; Xiao, H.-X.; Li, Y.; Zhou, T. MicroRNA-106b promotes colorectal cancer cell migration and invasion by directly targeting DLC1. *J. Exp. Clin. Cancer Res.* **2015**, *34*, 1–11, doi:10.1186/s13046-015-0189-7.
330. Liu, H.; Liu, Y.; Sun, P.; Leng, K.; Xu, Y.; Mei, L.; Han, P.; Zhang, B.; Yao, K.; Li, C.; et al. Colorectal cancer-derived exosomal miR-106b-3p promotes metastasis by down-regulating DLC-1 expression. *Clin. Sci.* **2020**, *134*, 419–434, doi:10.1042/cs20191087.
331. Wang, Y.-X.; Lang, F.; Liu, Y.-X.; Yang, C.-Q.; Gao, H.-J. In situ hybridization analysis of the expression of miR-106b in colonic cancer. *Int. J. Clin. Exp. Pathol.* **2015**, *8*, 786–792.
332. Ni, S.; Weng, W.; Xu, M.; Wang, Q.; Tan, C.; Sun, H.; Wang, L.; Huang, D.; Du, X.; Sheng, W. miR-106b-5p inhibits the invasion and metastasis of colorectal cancer by targeting CTSA. *OncoTargets Ther.* **2018**, *11*, 3835–3845, doi:10.2147/ott.s172887.
333. Chen, X.; Liu, J.; Zhang, Q.; Liu, B.; Cheng, Y.; Zhang, Y.; Sun, Y.; Ge, H.; Liu, Y. Exosome-mediated transfer of miR-93-5p from cancer-associated fibroblasts confer radioresistance in colorectal cancer cells by downregulating FOXA1 and upregulating TGFB3. *J. Exp. Clin. Cancer Res.* **2020**, *39*, 65–15, doi:10.1186/s13046-019-1507-2.
334. Marchi, S.; Lupini, L.; Patergnani, S.; Rimessi, A.; Missiroli, S.; Bonora, M.; Bononi, A.; Corrà, F.; Giorgi, C.; De Marchi, E.; et al. Downregulation of the Mitochondrial Calcium Uniporter by Cancer-Related miR-25. *Curr. Biol.* **2013**, *23*, 58–63, doi:10.1016/j.cub.2012.11.026.
335. Zeng, Z.; Li, Y.; Pan, Y.; Lan, X.; Song, F.; Sun, J.; Zhou, K.; Liu, X.; Ren, X.; Wang, F.; et al. Cancer-derived exosomal miR-25-3p promotes pre-metastatic niche formation by inducing vascular permeability and angiogenesis. *Nat. Commun.* **2018**, *9*, 1–14, doi:10.1038/s41467-018-07810-w.
336. Li, Q.; Zou, C.; Zou, C.; Han, Z.; Xiao, H.; Wei, H.; Wang, W.; Zhang, L.; Zhang, X.; Tang, Q.; et al. MicroRNA-25 functions as a potential tumor suppressor in colon cancer by targeting Smad7. *Cancer Lett.* **2013**, *335*, 168–174, doi:10.1016/j.canlet.2013.02.029.
337. Wang, D.; Wang, X.; Si, M.; Yang, J.; Sun, S.; Wu, H.; Cui, S.; Qu, X.; Yu, X. Exosome-encapsulated miRNAs contribute to CXCL12/CXCR4-induced liver metastasis of colorectal cancer by enhancing M2 polarization of macrophages. *Cancer Lett.* **2020**, *474*, 36–52, doi:10.1016/j.canlet.2020.01.005.
338. Luo, H.; Zou, J.; Dong, Z.; Zeng, Q.; Wu, D.; Liu, L. Up-regulated miR-17 promotes cell proliferation, tumour growth and cell cycle progression by targeting the RND3 tumour suppressor gene in colorectal carcinoma. *Biochem. J.* **2012**, *442*, 311–321, doi:10.1042/bj20111517.
339. Lu, D.; Tang, L.; Zhuang, Y.; Zhao, P. miR-17-3P regulates the proliferation and survival of colon cancer cells by targeting Par4. *Mol. Med. Rep.* **2017**, *17*, 618–623, doi:10.3892/mmr.2017.7863.
340. Liu, G.; Liu, Y.; Yang, Z.; Wang, J.; Li, D.; Zhang, X. Tumor suppressor microRNA-18a regulates tumor proliferation and invasion by targeting TBPL1 in colorectal cancer cells. *Mol. Med. Rep.* **2015**, *12*, 7643–7648, doi:10.3892/mmr.2015.4335.
341. Yin, Y.; Zhong, J.; Li, S.-W.; Li, J.-Z.; Zhou, M.; Chen, Y.; Sang, Y.; Liu, L. TRIM11, a direct target of miR-24-3p, promotes cell proliferation and inhibits apoptosis in colon cancer. *Oncotarget* **2016**, *7*, 86755–86765, doi:10.18632/oncotarget.13550.
342. Huang, L.; Wang, X.; Wen, C.; Yang, X.; Song, M.; Chen, J.; Wang, C.; Zhang, B.; Wang, L.; Iwamoto, A.; et al. Hsa-miR-19a is associated with lymph metastasis and mediates the TNF-α induced epithelial-to-mesenchymal transition in colorectal cancer. *Sci. Rep.* **2015**, *5*, 13350, doi:10.1038/srep13350.

343. Wang, T.; Xu, X.; Xu, Q.; Ren, J.; Shen, S.; Fan, C.; Hou, Y. miR-19a promotes colitis-associated colorectal cancer by regulating tumor necrosis factor alpha-induced protein 3-NF-κB feedback loops. *Oncogene* **2016**, *36*, 3240–3251, doi:10.1038/onc.2016.468.
344. Maminezhad, H.; Ghanadian, S.; Pakravan, K.; Razmara, E.; Rouhollah, F.; Mossahebi-Mohammadi, M.; Babashah, S. A panel of six-circulating miRNA signature in serum and its potential diagnostic value in colorectal cancer. *Life Sci.* **2020**, *258*, 118226, doi:10.1016/j.lfs.2020.118226.
345. Marcuello, M.; Duran-Sanchon, S.; Moreno, L.; Lozano, J.J.; Bujanda, L.; Castells, A.; Gironella, M. Analysis of A 6-Mirna Signature in Serum from Colorectal Cancer Screening Participants as Non-Invasive Biomarkers for Advanced Adenoma and Colorectal Cancer Detection. *Cancers* **2019**, *11*, 1542, doi:10.3390/cancers11101542.
346. Zhu, M.; Huang, Z.; Zhu, D.; Zhou, X.; Shan, X.; Qi, L.-W.; Wu, L.; Cheng, W.; Zhu, J.; Zhang, L.; et al. A panel of microRNA signature in serum for colorectal cancer diagnosis. *Oncotarget* **2017**, *8*, 17081–17091, doi:10.18632/oncotarget.15059.
347. Yu, F.-B.; Sheng, J.; Yu, J.-M.; Liu, J.-H.; Qin, X.-X.; Mou, B. MiR-19a-3p regulates the Forkhead box F2-mediated Wnt/β-catenin signaling pathway and affects the biological functions of colorectal cancer cells. *World J. Gastroenterol.* **2020**, *26*, 627–644, doi:10.3748/wjg.v26.i6.627.
348. Eslamizadeh, S.; Heidari, M.; Agah, S.; Faghihloo, E.; Ghazi, H.; Mirzaei, A.; Akbari, A. The Role of MicroRNA Signature as Diagnostic Biomarkers in Different Clinical Stages of Colorectal Cancer. *Cell J.* **2018**, *20*, 220–230.
349. Xu, T.; Jing, C.; Shi, Y.; Miao, R.; Peng, L.; Kong, S.; Ma, Y.; Li, L. microRNA-20a enhances the epithelial-to-mesenchymal transition of colorectal cancer cells by modulating matrix metalloproteinases. *Exp. Ther. Med.* **2015**, *10*, 683–688, doi:10.3892/etm.2015.2538.
350. Huang, G.; Chen, X.; Cai, Y.; Wang, X.; Xing, C. miR-20a-directed regulation of BID is associated with the TRAIL sensitivity in colorectal cancer. *Oncol. Rep.* **2016**, *37*, 571–578, doi:10.3892/or.2016.5278.
351. Zhu, H.; Ji, Y.; Li, W.; Wu, M. Identification of key pathways and genes in colorectal cancer to predict the prognosis based on mRNA interaction network. *Oncol. Lett.* **2019**, *18*, 3778–3786, doi:10.3892/ol.2019.10698.
352. Jin, H.; Fan, Y.; Yang, Q.; Wang, S.; Huang, J.; Xia, C. Serum miR-20a and miR-486 are potential biomarkers for discriminating colorectal neoplasia: A pilot study. *J. Cancer Res. Ther.* **2018**, *14*, 1572, doi:10.4103/jcrt.jcrt_1198_16.
353. Shi, Y.; Liu, Z. Serum miR-92a-1 is a novel diagnostic biomarker for colorectal cancer. *J. Cell. Mol. Med.* **2020**, *24*, 8363–8367, doi:10.1111/jcmm.15282.
354. Wang, J.; Huang, S.-K.; Zhao, M.; Yang, M.; Zhong, J.-L.; Gu, Y.-Y.; Peng, H.; Che, Y.-Q.; Huang, C.-Z. Identification of a Circulating MicroRNA Signature for Colorectal Cancer Detection. *PLoS ONE* **2014**, *9*, e87451, doi:10.1371/journal.pone.0087451.
355. Wei, Z.; Cui, L.; Mei, Z.; Liu, M.; Zhang, D. miR-181a mediates metabolic shift in colon cancer cells via the PTEN/AKT pathway. *FEBS Lett.* **2014**, *588*, 1773–1779, doi:10.1016/j.febslet.2014.03.037.
356. Liu, Y.; Guo, Y.; Liang, H.; Cheng, R.; Yang, F.; Hong, Y.; Zhao, C.; Liu, M.; Yu, M.; Zhou, X.; et al. miR-181b functions as an oncomiR in colorectal cancer by targeting PDCD4. *Protein Cell* **2016**, *7*, 722–734, doi:10.1007/s13238-016-0313-2.
357. Zhao, L.-D.; Zheng, W.-W.; Wang, G.-X.; Kang, X.-C.; Qin, L.; Ji, J.-J.; Hao, S. Epigenetic silencing of miR-181b contributes to tumorigenicity in colorectal cancer by targeting RASSF1A. *Int. J. Oncol.* **2016**, *48*, 1977–1984, doi:10.3892/ijo.2016.3414.
358. Chira, A.; Muresan, M.-S.; Braicu, C.; Budisan, L.; Raduly, L.; Chira, R.I.; Dumitrascu, D.L.; Berindan-Neagoe, I. Mir-23a and mir-181b serum levels in irritable bowel syndrome and colorectal cancer—A pilot study. *Bosn. J. Basic Med. Sci.* **2019**, *20*, 254–261, doi:10.17305/bjbms.2019.4392.
359. Yilmaz, N.; Yilmaz, U.; Tanbek, K.; Arikan, S.; Aksakal, N.; Zeybek, U.; Ergen, A. The role of miRNAs targeting K-ras and APC genes in colorectal cancer. *Bratisl. Med., J.* **2020**, *121*, 554–557, doi:10.4149/bll_2020_092.
360. Yuan, D.; Li, K.; Zhu, K.; Yan, R.; Dang, C. Plasma miR-183 predicts recurrence and prognosis in patients with colorectal cancer. *Cancer Biol. Ther.* **2015**, *16*, 268–275, doi:10.1080/15384047.2014.1002327.
361. Huangfu, L.; Liang, H.; Wang, G.; Su, X.; Li, L.; Du, Z.; Hu, M.; Dong, Y.; Bai, X.; Liu, T.; et al. miR-183 regulates autophagy and apoptosis in colorectal cancer through targeting of UVRAG. *Oncotarget* **2015**, *7*, 4735–4745, doi:10.18632/oncotarget.6732.
362. Wang, G.; Zhou, J.; Lu, F.; Qiu, L.; Xu, L.; Yang, X.; Miao, Y. Downregulation of microRNA-183-5p inhibits the proliferation and invasion of colorectal cancer cells by inactivating the reticulocalbin-2/Wnt/β-catenin signaling pathway. *Mol. Med. Rep.* **2019**, *19*, 4475–4483, doi:10.3892/mmr.2019.10059.
363. Ge, T.; Xiang, P.; Mao, H.; Tang, S.; Zhou, J.; Zhang, Y. Inhibition of miR-96 enhances the sensitivity of colorectal cancer cells to oxaliplatin by targeting TPM1. *Exp. Ther. Med.* **2020**, *20*, 2134–2140, doi:10.3892/etm.2020.8936.
364. Yang, M.-H.; Yu, J.; Jiang, D.-M.; Li, W.-L.; Wang, S.; Ding, Y.-Q. microRNA-182 targets special AT-rich sequence-binding protein 2 to promote colorectal cancer proliferation and metastasis. *J. Transl. Med.* **2014**, *12*, 109, doi:10.1186/1479-5876-12-109.
365. Liu, B.; Liu, Y.; Zhao, L.; Pan, Y.; Shan, Y.; Lifen, Z.; Yujia, S. Upregulation of microRNA-135b and microRNA-182 promotes chemoresistance of colorectal cancer by targeting ST6GALNAC2 via PI3K/AKT pathway. *Mol. Carcinog.* **2017**, *56*, 2669–2680, doi:10.1002/mc.22710.
366. Jin, Y.; Zhang, Z.-L.; Huang, Y.; Zhang, K.-N.; Xiong, B. MiR-182-5p inhibited proliferation and metastasis of colorectal cancer by targeting MTDH. *Eur. Rev. Med. Pharmacol. Sci.* **2019**, *23*, 1494–1501.
367. Al-Sheikh, Y.A.; Ghneim, H.K.; Alharbi, K.K.; Aboul-Soud, M.A. Screening for differentially-expressed microRNA biomarkers in Saudi colorectal cancer patients by small RNA deep sequencing. *Int. J. Mol. Med.* **2019**, *44*, 2027–2036, doi:10.3892/ijmm.2019.4362.
368. Xi, X.-P.; Zhuang, J.; Teng, M.-J.; Xia, L.-J.; Yang, M.-Y.; Liu, Q.-G.; Chen, J.-B. MicroRNA-17 induces epithelial-mesenchymal transition consistent with the cancer stem cell phenotype by regulating CYP7B1 expression in colon cancer. *Int. J. Mol. Med.* **2016**, *38*, 499–506, doi:10.3892/ijmm.2016.2624.

369. Zhang, X.-D.; Li, K.-K.; Gao, L.; Li, S.-Z.; Chen, K.; Zhang, J.-B.; Wang, D.; Tu, R.-F.; Zhang, J.-X.; Tao, K.-X.; et al. miR-191 promotes tumorigenesis of human colorectal cancer through targeting C/EBPβ. *Oncotarget* **2015**, *6*, 4144–4158, doi:10.18632/oncotarget.2864.
370. Hu, T.; Wu, X.; Li, K.; Li, Y.; He, P.; Wu, Z.; Jie, F.; Liu, W.; Guan, M. *AKAP12* Endogenous Transcripts Suppress The Proliferation, Migration And Invasion Of Colorectal Cancer Cells By Directly Targeting oncomiR-183-5p. *OncoTargets Ther.* **2019**, *12*, 8301–8310, doi:10.2147/OTT.S207600.
371. Ding, M.; Zhang, T.; Li, S.; Zhang, Y.; Qiu, Y.; Zhang, B. Correlation analysis between liver metastasis and serum levels of miR-200 and miR-141 in patients with colorectal cancer. *Mol. Med. Rep.* **2017**, *16*, 7791–7795, doi:10.3892/mmr.2017.7538.
372. Kim, B.-K.; Yoo, H.-I.; Lee, A.-R.; Choi, K.; Yoon, S.K. Decreased expression ofVLDLRis inversely correlated with miR-200c in human colorectal cancer. *Mol. Carcinog.* **2017**, *56*, 1620–1629, doi:10.1002/mc.22618.
373. Chen, J.; Wang, W.; Zhang, Y.; Chen, Y.; Hu, T. Predicting distant metastasis and chemoresistance using plasma miRNAs. *Med. Oncol.* **2013**, *31*, 1–7, doi:10.1007/s12032-013-0799-x.
374. Mazraehshah, M.K.; Tavangar, S.M.; Saidijam, M.; Amini, R.; Bahreini, F.; Dermani, F.K.; Najafi, R. Anticancer effects of miR-200c in colorectal cancer through BMI*J. Cell. Biochem.* **2018**, *119*, 10005–10012, doi:10.1002/jcb.27330.
375. Ye, J.; Wei, X.; Shang, Y.; Pan, Q.; Yang, M.; Tian, Y.; He, Y.; Peng, Z.; Chen, L.; Chen, W.; et al. Core 3 mucin-type O-glycan restoration in colorectal cancer cells promotes MUC1/p53/miR-200c-dependent epithelial identity. *Oncogene* **2017**, *36*, 6391–6407, doi:10.1038/onc.2017.241.
376. Bahnassy, A.A.; Salem, S.E.; El-Sayed, M.; Khorshid, O.; Lateif, M.A.; Youssef, A.S.; Mohanad, M.; Hussein, M.; Zekri, A.-R.; Ali, N.M. MiRNAs as molecular biomarkers in stage II egyptian colorectal cancer patients. *Exp. Mol. Pathol.* **2018**, *105*, 260–271, doi:10.1016/j.yexmp.2018.09.002.
377. Meneses, X.; Rios, R.; Huertas-Salgado, A.; Serrano, M.L. Circulating miR-141-3p, miR-143-3p and miR-200c-3p are differentially expressed in colorectal cancer and advanced adenomas. *Mol. Clin. Oncol.* **2019**, *11*, 201–207, doi:10.3892/mco.2019.1876.
378. Huang, G.; Wei, B.; Chen, Z.; Wang, J.; Zhao, L.; Peng, X.; Liu, K.; Lai, Y.; Ni, L. Identification of a four-microRNA panel in serum as promising biomarker for colorectal carcinoma detection. *Biomark. Med.* **2020**, *14*, 749–760, doi:10.2217/bmm-2019-0605.
379. Wang, J.; Yang, J.; Zhang, H.; Liao, Y.; Xu, D.; Ma, S. Effects of miR-135a-5p and miR-141 on proliferation, invasion and apoptosis of colorectal cancer SW620 cells. *Oncol. Lett.* **2020**, *20*, 914–920, doi:10.3892/ol.2020.11598.
380. Ding, L.; Yu, L.-L.; Han, N.; Zhang, B.-T. miR-141 promotes colon cancer cell proliferation by inhibiting MAP2K4. *Oncol. Lett.* **2017**, *13*, 1665–1671, doi:10.3892/ol.2017.5653.
381. Wu, P.P.; Zhu, H.Y.; Sun, X.F.; Chen, L.X.; Zhou, Q.; Chen, J. MicroRNA-141 regulates the tumour suppressor DLC1 in colorectal cancer. *Neoplasma* **2015**, *62*, 705–712, doi:10.4149/neo_2015_084.
382. Feng, L.; Ma, H.; Chang, L.; Zhou, X.; Wang, N.; Zhao, L.; Zuo, J.; Wang, Y.; Han, J.; Wang, G. Role of microRNA-141 in colorectal cancer with lymph node metastasis. *Exp. Ther. Med.* **2016**, *12*, 3405–3410, doi:10.3892/etm.2016.3751.
383. Peng, Z.; Zhu, W.; Dai, J.; Ju, F. MicroRNA-200 as potential diagnostic markers for colorectal cancer: Meta-analysis and experimental validation. *Cell. Mol. Biol.* **2018**, *64*, 77–85, doi:10.14715/cmb/2018.64.6.14.
384. Chen, L.; Gao, H.; Liang, J.; Qiao, J.; Duan, J.; Shi, H.; Zhen, T.; Li, H.; Zhang, F.; Zhu, Z.; et al. miR-203a-3p promotes colorectal cancer proliferation and migration by targeting PDE4D. *Am. J. Cancer Res.* **2018**, *8*, 2387–2401.
385. To, K.K.; Leung, W.W.; Ng, S.S. A novel miR-203-DNMT3b-ABCG2 regulatory pathway predisposing colorectal cancer development. *Mol. Carcinog.* **2016**, *56*, 464–477, doi:10.1002/mc.22508.
386. Fu, Q.; Zhang, J.; Xu, X.; Qian, F.; Feng, K.; Ma, J. miR-203 is a predictive biomarker for colorectal cancer and its expression is associated with BIRC5. *Tumor Biol.* **2016**, *37*, 15989–15995, doi:10.1007/s13277-016-5438-7.
387. Liu, Y.; Gao, S.; Chen, X.; Liu, M.; Mao, C.; Fang, X. Overexpression of miR-203 sensitizes paclitaxel (Taxol)-resistant colorectal cancer cells through targeting the salt-inducible kinase 2 (SIK2). *Tumor Biol.* **2016**, *37*, 12231–12239, doi:10.1007/s13277-016-5066-2.
388. Wang, Y.-N.; Chen, Z.-H.; Chen, W.-C. Novel circulating microRNAs expression profile in colon cancer: A pilot study. *Eur. J. Med. Res.* **2017**, *22*, 1–11, doi:10.1186/s40001-017-0294-5.
389. Orosz, E.; Kiss, I.; Gyöngyi, Z.; Varjas, T. Expression of Circulating miR-155, miR-21, miR-221, miR-30a, miR-34a and miR-29a: Comparison of Colonic and Rectal Cancer. *Vivo* **2018**, *32*, 1333–1337, doi:10.21873/invivo.11383.
390. Farace, C.; Pisano, A.; Griñan-Lison, C.; Solinas, G.; Jiménez, G.; Serra, M.; Carrillo, E.; Scognamillo, F.; Attene, F.; Montella, A.; et al. Deregulation of cancer-stem-cell-associated miRNAs in tissues and sera of colorectal cancer patients. *Oncotarget* **2020**, *11*, 116–130, doi:10.18632/oncotarget.27411.
391. Yong, F.L.; Law, C.W.; Wang, C.W. Potentiality of a triple microRNA classifier: miR-193a-3p, miR-23a and miR-338-5p for early detection of colorectal cancer. *BMC Cancer* **2013**, *13*, 280, doi:10.1186/1471-2407-13-280.
392. Yong, F.L.; Wang, C.W.; Roslani, A.C.; Law, C.W. The Involvement of miR-23a/APAF1 Regulation Axis in Colorectal Cancer. *Int. J. Mol. Sci.* **2014**, *15*, 11713–11729, doi:10.3390/ijms150711713.
393. Tang, X.; Yang, M.; Wang, Z.; Wu, X.; Wang, D. MicroRNA-23a promotes colorectal cancer cell migration and proliferation by targeting at MARK1. *Acta Biochim. Biophys. Sin.* **2019**, *51*, 661–668, doi:10.1093/abbs/gmz047.
394. Jin, F.; Yang, R.; Wei, Y.; Wang, D.; Zhu, Y.; Wang, X.; Lu, Y.; Wang, Y.; Zen, K.; Li, L. HIF-1α-induced miR-23a~27a~24 cluster promotes colorectal cancer progression via reprogramming metabolism. *Cancer Lett.* **2019**, *440-441*, 211–222, doi:10.1016/j.canlet.2018.10.025.
395. Qin, D.; Wei, R.; Liu, S.; Zhu, S.; Zhang, S.; Min, L. A Circulating miRNA-Based Scoring System Established by WGCNA to Predict Colon Cancer. *Anal. Cell. Pathol.* **2019**, *2019*, 1–7, doi:10.1155/2019/1571045.

396. Maqbool, R.; Lone, S.N.; Hussain, M.U. Post-transcriptional regulation of the tumor suppressor p53 by a novel miR-27a, with implications during hypoxia and tumorigenesis. *Biochem. J.* **2016**, *473*, 3597–3610, doi:10.1042/bcj20160359.
397. Tan, Y.; Lin, J.-J.; Yang, X.; Gou, D.-M.; Fu, L.; Li, F.-R.; Yu, X.-F. A panel of three plasma microRNAs for colorectal cancer diagnosis. *Cancer Epidemiol.* **2019**, *60*, 67–76, doi:10.1016/j.canep.2019.01.015.
398. He, H.; Wang, N.; Yi, X.; Tang, C.; Wang, D. Low-level serum miR-24-2 is associated with the progression of colorectal cancer. *Cancer Biomark.* **2018**, *21*, 261–267, doi:10.3233/cbm-170321.
399. Hao, J.-P.; Ma, A. The ratio of miR-21/miR-24 as a promising diagnostic and poor prognosis biomarker in colorectal cancer. *Eur. Rev. Med. Pharmacol. Sci.* **2018**, *22*, 8649–8656.
400. Fang, Z.; Tang, J.; Bai, Y.; Lin, H.; You, H.; Jin, H.; Lin, L.; You, P.; Li, J.; Dai, Z.; et al. Plasma levels of microRNA-24, microRNA-320a, and microRNA-423-5p are potential biomarkers for colorectal carcinoma. *J. Exp. Clin. Cancer Res.* **2015**, *34*, 1–10, doi:10.1186/s13046-015-0198-6.
401. Wang, B.; Li, W.; Liu, H.; Yang, L.; Liao, Q.; Cui, S.; Wang, H.; Zhao, L. miR-29b suppresses tumor growth and metastasis in colorectal cancer via downregulating Tiam1 expression and inhibiting epithelial–mesenchymal transition. *Cell Death Dis.* **2014**, *5*, e1335, doi:10.1038/cddis.2014.304.
402. Basati, G.; Razavi, A.E.; Pakzad, I.; Malayeri, F.A. Circulating levels of the miRNAs, miR-194, and miR-29b, as clinically useful biomarkers for colorectal cancer. *Tumor Biol.* **2015**, *37*, 1781–1788, doi:10.1007/s13277-015-3967-0.
403. He, P.Y.; Yip, W.K.; Chai, B.; Jabar, M.F.; Dusa, N.; Mohtarrudin, N.; Seow, H.F. Inhibition of cell migration and invasion by miR?29a?3p in a colorectal cancer cell line through suppression of CDC42BPA mRNA expression. *Oncol. Rep.* **2017**, *38*, 3554–3566,, doi:10.3892/or.2017.6037.
404. Zheng, Z.; Cui, H.; Wang, Y.; Yao, W. Downregulation of RPS15A by miR-29a-3p attenuates cell proliferation in colorectal carcinoma. *Biosci. Biotechnol. Biochem.* **2019**, *83*, 2057–2064, doi:10.1080/09168451.2019.1637712.
405. Yamada, A.; Horimatsu, T.; Okugawa, Y.; Nishida, N.; Honjo, H.; Ida, H.; Kou, T.; Kusaka, T.; Sasaki, Y.; Yagi, M.; et al. Serum miR-21, miR-29a, and miR-125b Are Promising Biomarkers for the Early Detection of Colorectal Neoplasia. *Clin. Cancer Res.* **2015**, *21*, 4234–4242, doi:10.1158/1078-0432.ccr-14-2793.
406. Yi, R.; Li, Y.; Wang, F.; Gu, J.; Isaji, T.; Li, J.; Qi, R.; Zhu, X.; Zhao, Y. Transforming growth factor (TGF) β1 acted through miR-130b to increase integrin α5 to promote migration of colorectal cancer cells. *Tumor Biol.* **2016**, *37*, 10763–10773, doi:10.1007/s13277-016-4965-6.
407. Fassan, M.; Cui, R.; Gasparini, P.; Mescoli, C.; Guzzardo, V.; Vicentini, C.; Munari, G.; Loupakis, F.; Lonardi, S.; Braconi, C.; et al. miR-224 Is Significantly Upregulated and Targets Caspase-3 and Caspase-7 During Colorectal Carcinogenesis. *Transl. Oncol.* **2019**, *12*, 282–291, doi:10.1016/j.tranon.2018.10.013.
408. Angius, A.; Uva, P.; Pira, G.; Muroni, M.R.; Sotgiu, G.; Saderi, L.; Uleri, E.; Caocci, M.; Ibba, G.; Cesaraccio, M.R.; et al. Integrated Analysis of miRNA and mRNA Endorses a Twenty miRNAs Signature for Colorectal Carcinoma. *Int. J. Mol. Sci.* **2019**, *20*, 4067, doi:10.3390/ijms20164067.
409. Liao, W.-T.; Li, T.-T.; Wang, Z.-G.; Wang, S.-Y.; He, M.-R.; Ye, Y.-P.; Qi, L.; Cui, Y.-M.; Wu, P.; Jiao, H.-L.; et al. microRNA-224 Promotes Cell Proliferation and Tumor Growth in Human Colorectal Cancer by Repressing PHLPP1 and PHLPP2. *Clin. Cancer Res.* **2013**, *19*, 4662–4672, doi:10.1158/1078-0432.ccr-13-0244.
410. Ke, T.-W.; Hsu, H.-L.; Wu, Y.-H.; Chen, W.T.-L.; Cheng, Y.-W.; Cheng, C.-W. MicroRNA-224 Suppresses Colorectal Cancer Cell Migration by Targeting Cdc42. *Dis. Markers* **2014**, *2014*, 1–11, doi:10.1155/2014/617150.
411. Feng, B.; Dong, T.T.; Wang, L.L.; Zhou, H.M.; Zhao, H.C.; Dong, F.; Zheng, M.H. Colorectal Cancer Migration and Invasion Initiated by microRNA-106a. *PLoS ONE* **2012**, *7*, e43452, doi:10.1371/journal.pone.0043452.
412. Lai, H.; Zhang, J.; Zuo, H.; Liu, H.; Xu, J.; Feng, Y.; Lin, Y.; Mo, X. Overexpression of miR-17 is correlated with liver metastasis in colorectal cancer. *Med.* **2020**, *99*, e19265, doi:10.1097/md.0000000000019265.
413. Kim, T.W.; Lee, Y.S.; Yun, N.H.; Shin, C.H.; Hong, H.K.; Kim, H.H.; Cho, Y.B. MicroRNA-17-5p regulates EMT by targeting vimentin in colorectal cancer. *Br. J. Cancer* **2020**, *123*, 1–8, doi:10.1038/s41416-020-0940-5.
414. Wikberg, M.L.; Myte, R.; Palmqvist, R.; Van Guelpen, B.; Ljuslinder, I. Plasma miRNA can detect colorectal cancer, but how early? *Cancer Med.* **2018**, *7*, 1697–1705, doi:10.1002/cam4.1398.
415. Cheng, D.; Zhao, S.; Tang, H.; Zhang, D.; Sun, H.; Yu, F.; Jiang, W.; Yue, B.; Wang, J.; Zhang, M.; et al. MicroRNA-20a-5p promotes colorectal cancer invasion and metastasis by downregulating Smad4. *Oncotarget* **2016**, *7*, 45199–45213, doi:10.18632/oncotarget.9900.
416. Zhang, G.; Zhou, H.; Xiao, H.; Liu, Z.; Tian, H.; Zhou, T. MicroRNA-92a Functions as an Oncogene in Colorectal Cancer by Targeting PTEN. *Dig. Dis. Sci.* **2014**, *59*, 98–107, doi:10.1007/s10620-013-2858-8.
417. Qin, S.; Zhu, Y.; Ai, F.; Li, Y.; Bai, B.; Yao, W.; Dong, L. MicroRNA-191 correlates with poor prognosis of colorectal carcinoma and plays multiple roles by targeting tissue inhibitor of metalloprotease *Neoplasma* **2014**, *61*, 27–34, doi:10.4149/neo_2014_005.
418. Lu, Y.-X.; Yuan, L.; Xue, X.-L.; Zhou, M.; Liu, Y.; Zhang, C.; Li, J.-P.; Zheng, L.; Hong, M.; Li, X.-N. Regulation of Colorectal Carcinoma Stemness, Growth, and Metastasis by an miR-200c-Sox2–Negative Feedback Loop Mechanism. *Clin. Cancer Res.* **2014**, *20*, 2631–2642, doi:10.1158/1078-0432.ccr-13-2348.
419. Muto, Y.; Suzuki, K.; Kato, T.; Tsujinaka, S.; Ichida, K.; Takayama, Y.; Fukui, T.; Kakizawa, N.; Watanabe, F.; Saito, M.; et al. Heterogeneous expression of zinc-finger E-box-binding homeobox 1 plays a pivotal role in metastasis via regulation of miR-200c in epithelial-mesenchymal transition. *Int. J. Oncol.* **2016**, *49*, 1057–1067, doi:10.3892/ijo.2016.3583.
420. Zhang, W.-W.; Ming, X.-L.; Rong, Y.; Huang, C.-Q.; Weng, H.; Chen, H.; Bian, J.-M.; Wang, F.-B. Diagnostic Value Investigation and Bioinformatics Analysis of miR-31 in Patients with Lymph Node Metastasis of Colorectal Cancer. *Anal. Cell. Pathol.* **2019**, *2019*, 1–10, doi:10.1155/2019/9740475.

421. Yin, J.; Bai, Z.-G.; Song, J.; Yang, Y.; Wang, J.; Han, W.; Zhang, J.; Meng, H.; Ma, X.; Yang, Y.; et al. Differential expression of serum miR-126, miR-141 and miR-21 as novel biomarkers for early detection of liver metastasis in colorectal cancer. *Chin. J. Cancer Res.* **2014**, *26*, 95–103, doi:10.3978/j.issn.1000-9604.2014.02.07.
422. Miao, J.; Hou, N.; Yang, W.; Jiang, Q.; Xue, W.; Wang, X.; Zhang, H.; Xiong, X.; Wang, L.; Zhao, L.; et al. miR-203a suppresses cell proliferation by targeting RING-finger protein 6 in colorectal cancer. *Anti-Cancer Drugs* **2020**, *31*, 583–591, doi:10.1097/cad.0000000000000874.
423. Cui, Q. Significance of miR-27a and miR-31 in early diagnosis and prognosis of colorectal cancer. *Oncol. Lett.* **2019**, *18*, 3092–3096, doi:10.3892/ol.2019.10621.
424. Faltejskova, P.; Bocanek, O.; Sachlova, M.; Svoboda, M.; Kiss, I.; Vyzula, R.; Slaby, O. Circulating miR-17-3p, miR-29a, miR-92a and miR-135b in serum: Evidence against their usage as biomarkers in colorectal cancer. *Cancer Biomark.* **2013**, *12*, 199–204, doi:10.3233/cbm-130308.
425. Wang, L.-G.; Gu, J. Serum microRNA-29a is a promising novel marker for early detection of colorectal liver metastasis. *Cancer Epidemiol.* **2012**, *36*, e61–e67, doi:10.1016/j.canep.2011.05.002.
426. Bi, D.-P.; Yin, C.-H.; Zhang, X.-Y.; Yang, N.-N.; Xu, J.-Y. miR-183 functions as an oncogene by targeting ABCA1 in colon cancer. *Oncol. Rep.* **2016**, *35*, 2873–2879, doi:10.3892/or.2016.4631.
427. Li, X.; Li, X.; Liao, D.; Wang, X.; Wu, Z.; Nie, J.; Bai, M.; Fu, X.; Mei, Q.; Han, W. Elevated microRNA-23a Expression Enhances the Chemoresistance of Colorectal Cancer Cells with Microsatellite Instability to 5-Fluorouracil by Directly Targeting ABCF-*Curr. Protein Pept. Sci.* **2015**, *16*, 301–309, doi:10.2174/1389203716604150429153309.
428. Pichler, M.; Stiegelbauer, V.; Vychytilova-Faltejskova, P.; Ivan, C.; Ling, H.; Winter, E.; Zhang, X.; Goblirsch, M.; Wulf-Goldenberg, A.; Ohtsuka, M.; et al. Genome-Wide miRNA Analysis Identifies miR-188-3p as a Novel Prognostic Marker and Molecular Factor Involved in Colorectal Carcinogenesis. *Clin. Cancer Res.* **2016**, *23*, 1323–1333, doi:10.1158/1078-0432.CCR-16-0497.
429. Li, J.; Chen, Y.; Zhao, J.; Kong, F.; Zhang, Y. miR-203 reverses chemoresistance in p53-mutated colon cancer cells through downregulation of Akt2 expression. *Cancer Lett.* **2011**, *304*, 52–59, doi:10.1016/j.canlet.2011.02.003.
430. Zhou, J.; Wang, J.; Wu, S.; Zhu, S.; Wang, S.; Zhou, H.; Tian, X.; Tang, N.; Nie, S. Angiopoietin-like protein 2 negatively regulated by microRNA-25 contributes to the malignant progression of colorectal cancer. *Int. J. Mol. Med.* **2014**, *34*, 1286–1292, doi:10.3892/ijmm.2014.1909.
431. Zhang, H.; Du, W.B.; Guo, X.M.; Wang, L.K.; Cheng, J.M.; Wei, L.J. Identification of the AQP8-miR-92a network associated with the aggressive traits of colorectal cancer. *Biochem. Biophys. Res. Commun.* **2020**, *527*, 218–225, doi:10.1016/j.bbrc.2020.04.055.
432. Zhai, Z.; Wu, F.; Chuang, A.Y.; Kwon, J.H. miR-106b Fine Tunes ATG16L1 Expression and Autophagic Activity in Intestinal Epithelial HCT116 Cells. *Inflamm. Bowel Dis.* **2013**, *19*, 2295–2301, doi:10.1097/mib.0b013e31829e71cf.
433. Che, J.; Wang, W.; Huang, Y.; Zhang, L.; Zhao, J.; Zhang, P.; Yuan, X. miR-20a inhibits hypoxia-induced autophagy by targeting ATG5/FIP200 in colorectal cancer. *Mol. Carcinog.* **2019**, *58*, 1234–1247, doi:10.1002/mc.23006.
434. Tsuchida, A.; Ohno, S.; Wu, W.; Borjigin, N.; Fujita, K.; Aoki, T.; Ueda, S.; Takanashi, M.; Kuroda, M. miR-92 is a key oncogenic component of the miR-17-92 cluster in colon cancer. *Cancer Sci.* **2011**, *102*, 2264–2271, doi:10.1111/j.1349-7006.2011.02081.x.
435. Subramanian, M.; Rao, S.R.; Thacker, P.; Chatterjee, S.; Karunagaran, D. MiR-29b downregulates canonical Wnt signaling by targeting BCL9L and other coactivators of β-catenin in human colorectal cancer cells. *J. Cell. Biochem.* **2014**, *115*, 1974–1984, doi:10.1002/jcb.24869.
436. Chai, H.; Liu, M.; Tian, R.; Li, X.; Tang, H. miR-20a targets BNIP2 and contributes chemotherapeutic resistance in colorectal adenocarcinoma SW480 and SW620 cell lines. *Acta Biochim. et Biophys. Sin.* **2011**, *43*, 217–225, doi:10.1093/abbs/gmq125.
437. Su, C.; Huang, D.; Liu, J.; Liu, W.; Cao, Y. miR-27a-3p regulates proliferation and apoptosis of colon cancer cells by potentially targeting BTG1. *Oncol. Lett.* **2019**, *18*, 2825–2834, doi:10.3892/ol.2019.10629.
438. Colangelo, T.; Polcaro, G.; Ziccardi, P.; Pucci, B.; Muccillo, L.; Galgani, M.; Fucci, A.; Milone, M.R.; Budillon, A.; Santopaolo, M.; et al. Proteomic screening identifies calreticulin as a miR-27a direct target repressing MHC class I cell surface exposure in colorectal cancer. *Cell Death Dis.* **2016**, *7*, e2120, doi:10.1038/cddis.2016.28.
439. Humphreys, K.J.; Cobiac, L.; Le Leu, R.K.; Van der Hoek, M.B.; Michael, M.Z. Histone deacetylase inhibition in colorectal cancer cells reveals competing roles for members of the oncogenic miR-17-92 cluster. *Mol. Carcinog.* **2013**, *52*, 459–474, doi:10.1002/mc.21879.
440. Mencia, N.; Selga, E.; Noe, V.; Ciudad, C.J. Underexpression of miR-224 in methotrexate resistant human colon cancer cells. *Biochem. Pharmacol.* **2011**, *82*, 1572–1582, doi:10.1016/j.bcp.2011.08.009.
441. Zhang, H.; Guo, J.; Mao, L.; Li, Q.; Guo, M.; Mu, T.; Zhang, Q.; Bi, X. Up-regulation of miR-24-1-5p is involved in the chemoprevention of colorectal cancer by black raspberry anthocyanins. *Br. J. Nutr.* **2018**, *122*, 518–526, doi:10.1017/s0007114518003136.
442. Liu, D.; Zhang, H.; Cui, M.; Chen, C.; Feng, Y. Hsa-miR-425-5p promotes tumor growth and metastasis by activating the CTNND1-mediated β-catenin pathway and EMT in colorectal cancer. *Cell Cycle* **2020**, *19*, 1917–1927, doi:10.1080/15384101.2020.1783058.
443. Signs, S.A.; Fisher, R.C.; Tran, U.; Chakrabarti, S.; Sarvestani, S.K.; Xiang, S.; Liska, D.; Roche, V.; Lai, W.; Gittleman, H.R.; et al. Stromal miR-20a controls paracrine CXCL8 secretion in colitis and colon cancer. *Oncotarget* **2018**, *9*, 13048–13059, doi:10.18632/oncotarget.24495.
444. Mishra, P.J.; Song, B.; Mishra, P.J.; Wang, Y.; Humeniuk, R.; Banerjee, D.; Merlino, G.; Ju, J.; Bertino, J.R. MiR-24 Tumor Suppressor Activity Is Regulated Independent of p53 and through a Target Site Polymorphism. *PLoS ONE* **2009**, *4*, e8445, doi:10.1371/journal.pone.0008445.

445. Zhang, Q.; Li, W.; Liu, G.; Tang, W. MicroRNA-24 regulates the growth and chemosensitivity of the human colorectal cancer cells by targeting RNA-binding protein DND1. *J. B.U.ON* **2019**, *24*, 1476–1481.
446. Yu, C.; Sun, J.; Leng, X.; Yang, J. Long noncoding RNA SNHG6 functions as a competing endogenous RNA by sponging miR-181a-5p to regulate E2F5 expression in colorectal cancer. *Cancer Manag. Res.* **2019**, *11*, 611–624, doi:10.2147/cmar.s182719.
447. Xing, Y.; Jing, H.; Zhang, Y.; Suo, J.; Qian, M. MicroRNA-141-3p affected proliferation, chemosensitivity, migration and invasion of colorectal cancer cells by targeting EGFR. *Int. J. Biochem. Cell Biol.* **2020**, *118*, 105643, doi:10.1016/j.biocel.2019.105643.
448. Li, L.; Sarver, A.L.; Khatri, R.; Hajeri, P.B.; Kamenev, I.; French, A.J.; Thibodeau, S.N.; Steer, C.J.; Subramanian, S. Sequential expression of miR-182 and miR-503 cooperatively targets FBXW7, contributing to the malignant transformation of colon adenoma to adenocarcinoma. *J. Pathol.* **2014**, *234*, 488–501, doi:10.1002/path.4407.
449. Zhang, Y.; Wang, X.; Wang, Z.; Tang, H.; Fan, H.; Guo, Q. miR-182 promotes cell growth and invasion by targeting forkhead box F2 transcription factor in colorectal cancer. *Oncol. Rep.* **2015**, *33*, 2592–2598, doi:10.3892/or.2015.3833.
450. Qiang, Y.; Feng, L.; Wang, G.; Liu, J.; Zhang, J.; Xiang, L.; Su, C.; Zhang, S.; Xie, X.; Chen, E. miR-20a/Foxj2 Axis Mediates Growth and Metastasis of Colorectal Cancer Cells as Identified by Integrated Analysis. *Med. Sci. Monit.* **2020**, *26*, e923559, doi:10.12659/msm.923559.
451. Liu, Z.; Qin, Y.; Dong, S.; Chen, X.; Huo, Z.; Zhen, Z. Overexpression of miR-106a enhances oxaliplatin sensitivity of colorectal cancer through regulation of FOXQ1. *Oncol. Lett.* **2019**, *19*, 663–670, doi:10.3892/ol.2019.11151.
452. Longqiu, Y.; Pengcheng, L.; Xuejie, F.; Peng, Z. A mi RNA s panel promotes the proliferation and invasion of colorectal cancer cells by targeting GABBR *Cancer Med.* **2016**, *5*, 2022–2031, doi:10.1002/cam4.760.
453. Lai, H.; Tseng, W.; Huang, S.; Chao, T.; Su, Y. MicroRNA-203 diminishes the stemness of human colon cancer cells by suppressing GATA6 expression. *J. Cell. Physiol.* **2020**, *235*, 2866–2880, doi:10.1002/jcp.29192.
454. Hu, M.; Xia, M.; Chen, X.; Lin, Z.; Xu, Y.; Ma, Y.; Su, L. MicroRNA-141 Regulates Smad Interacting Protein 1 (SIP1) and Inhibits Migration and Invasion of Colorectal Cancer Cells. *Dig. Dis. Sci.* **2009**, *55*, 2365–2372, doi:10.1007/s10620-009-1008-9.
455. Fujiya, M.; Konishi, H.; Kamel, M.K.M.; Ueno, N.T.; Inaba, Y.; Moriichi, K.; Tanabe, H.; Ikuta, K.; Ohtake, T.; Kohgo, Y. microRNA-18a induces apoptosis in colon cancer cells via the autophagolysosomal degradation of oncogenic heterogeneous nuclear ribonucleoprotein A1. *Oncogene* **2014**, *33*, 4847–4856, doi:10.1038/onc.2013.429.
456. Zhao, Y.; Miao, G.; Li, Y.; Isaji, T.; Gu, J.; Li, J.; Qi, R. Microrna 130b Suppresses Migration and Invasion of Colorectal Cancer Cells through Downregulation of Integrin β. *PLoS ONE* **2014**, *9*, e87938, doi:10.1371/journal.pone.0087938.
457. Wang, Z.-Z.; Yang, J.; Jiang, B.-H.; Di, J.-B.; Gao, P.; Peng, L.; Su, X.-Q. KIF14 promotes cell proliferation via activation of Akt and is directly targeted by miR-200c in colorectal cancer. *Int. J. Oncol.* **2018**, *53*, 1939–1952, doi:10.3892/ijo.2018.4546.
458. Sells, E.; Pandey, R.; Chen, H.; Skovan, B.A.; Cui, H.; Ignatenko, N.A. Specific microRNA–mRNA Regulatory Network of Colon Cancer Invasion Mediated by Tissue Kallikrein–Related Peptidase 6. *Neoplasia* **2017**, *19*, 396–411, doi:10.1016/j.neo.2017.02.003.
459. Zhang, L.; He, L.; Zhang, H.; Chen, Y. Knockdown of MiR-20a Enhances Sensitivity of Colorectal Cancer Cells to Cisplatin by Increasing ASK1 Expression. *Cell. Physiol. Biochem.* **2018**, *47*, 1432–1441, doi:10.1159/000490834.
460. Wang, F.; Zhao, L.; Zhang, J.; Meng, Z.; Zhou, C.; Wang, G.; Liu, Y.; Li, M.; Xi, J.; Niu, W.; et al. Chemotherapy-induced miR-141/MAP4K4 signaling suppresses progression of colorectal cancer. *Biosci. Rep.* **2018**, *38*, doi:10.1042/bsr20180978.
461. Tang, S.; Fu, H.; Xu, Q.; Zhou, Y. miR-20a regulates sensitivity of colorectal cancer cells to NK cells by targeting MICA. *Biosci. Rep.* **2019**, *39*, doi:10.1042/bsr20180695.
462. Li, Y.; Kuscu, C.; Banach, A.; Zhang, Q.; Pulkoski-Gross, A.; Kim, D.; Liu, J.; Roth, E.; Li, E.; Shroyer, K.R.; et al. miR-181a-5p Inhibits Cancer Cell Migration and Angiogenesis via Downregulation of Matrix Metalloproteinase-14. *Cancer Res.* **2015**, *75*, 2674–2685, doi:10.1158/0008-5472.can-14-2875.
463. Jung, J.H.; Shin, E.A.; Kim, J.-H.; Sim, D.Y.; Lee, H.; Park, J.E.; Lee, H.-J.; Kim, S.-H. NEDD9 Inhibition by miR-25-5p Activation Is Critically Involved in Co-Treatment of Melatonin- and Pterostilbene-Induced Apoptosis in Colorectal Cancer Cells. *Cancers* **2019**, *11*, 1684, doi:10.3390/cancers11111684.
464. Han, R.; Sun, Q.; Wu, J.; Zheng, P.; Zhao, G. Sodium Butyrate Upregulates miR-203 Expression to Exert Anti-Proliferation Effect on Colorectal Cancer Cells. *Cell. Physiol. Biochem.* **2016**, *39*, 1919–1929, doi:10.1159/000447889.
465. Alcantara, K.M.M.; Garcia, R.L. MicroRNA-92a promotes cell proliferation, migration and survival by directly targeting the tumor suppressor gene NF2 in colorectal and lung cancer cells. *Oncol. Rep.* **2019**, *41*, 2103–2116, doi:10.3892/or.2019.7020.
466. Zhang, Y.; Hu, X.; Miao, X.; Zhu, K.; Cui, S.; Meng, Q.; Sun, J.; Wang, T. Micro RNA -425-5p regulates chemoresistance in colorectal cancer cells via regulation of Programmed Cell Death. *J. Cell. Mol. Med.* **2016**, *20*, 360–369, doi:10.1111/jcmm.12742.
467. Deng, Y.H.; Deng, Z.H.; Hao, H.; Wu, X.L.; Gao, H.; Tang, S.H.; Tang, H. MicroRNA-23a promotes colorectal cancer cell survival by targeting PDK4. *Exp. Cell Res.* **2018**, *373*, 171–179, doi:10.1016/j.yexcr.2018.10.010.
468. Huang, G.; Wu, X.; Li, S.; Xu, X.; Zhu, H.; Chen, X. The long noncoding RNA CASC2 functions as a competing endogenous RNA by sponging miR-18a in colorectal cancer. *Sci. Rep.* **2016**, *6*, 26524, doi:10.1038/srep26524.
469. Pan, X.; Feng, J.; Zhu, Z.; Yao, L.; Ma, S.; Hao, B.; Zhang, G. A positive feedback loop between miR-181b and STAT 3 that affects Warburg effect in colon cancer via regulating PIAS 3 expression. *J. Cell. Mol. Med.* **2018**, *22*, 5040–5049, doi:10.1111/jcmm.13786.
470. Shi, L.; Li, X.; Wu, Z.; Li, X.; Nie, J.; Guo, M.; Mei, Q.; Han, W. DNA methylation-mediated repression of miR-181a/135a/302c expression promotes the microsatellite-unstable colorectal cancer development and 5-FU resistance via targeting PLAG1. *J. Genet. Genom.* **2018**, *45*, 205–214, doi:10.1016/j.jgg.2018.04.003.
471. Ou, B.; Sun, H.; Zhao, J.; Xu, Z.; Liu, Y.; Feng, H.; Peng, Z. Polo-like kinase 3 inhibits glucose metabolism in colorectal cancer by targeting HSP90/STAT3/HK2 signaling. *J. Exp. Clin. Cancer Res.* **2019**, *38*, 1–12, doi:10.1186/s13046-019-1418-2.

472. Wang, H.; Cai, X.; Ma, L. Curcumin Modifies Epithelial–Mesenchymal Transition in Colorectal Cancer Through Regulation of miR-200c/EPM5. *Cancer Manag. Res.* **2020**, *12*, 9405–9415, doi:10.2147/cmar.s260129.
473. Zhang, S.; Zhang, Y.; Cheng, Q.; Ma, Z.; Gong, G.; Deng, Z.; Xu, K.; Wang, G.; Wei, Y.; Zou, X. Silencing protein kinase C ζ by microRNA-25-5p activates AMPK signaling and inhibits colorectal cancer cell proliferation. *Oncotarget* **2017**, *8*, 65329–65338, doi:10.18632/oncotarget.18649.
474. Qin, Y.; Huo, Z.; Song, X.; Chen, X.; Tian, X.; Wang, X. mir-106a regulates cell proliferation and apoptosis of colon cancer cells through targeting the PTEN/PI3K/AKT signaling pathway. *Oncol. Lett.* **2017**, *15*, 3197–3201, doi:10.3892/ol.2017.7715.
475. Zhang, Y.; Liu, X.; Zhang, J.; Xu, Y.; Shao, J.; Hu, Y.; Shu, P.; Cheng, H. Inhibition of miR-19a partially reversed the resistance of colorectal cancer to oxaliplatin via PTEN/PI3K/AKT pathway. *Aging* **2020**, *12*, 5640–5650, doi:10.18632/aging.102929.
476. Yuan, L.-L.; Li, L.; Liu, J.-N.; Mei, J.; Lei, C.-J. Down-regulation of miR-29a facilitates apoptosis of colorectal carcinoma cell SW480 and suppresses its Paclitaxel resistance. *Eur. Rev. Med. Pharmacol. Sci.* **2018**, *22*, 5499–5507.
477. Iseki, Y.; Shibutani, M.; Maeda, K.; Nagahara, H.; Fukuoka, T.; Matsutani, S.; Hirakawa, K.; Ohira, M. MicroRNA-96 Promotes Tumor Invasion in Colorectal Cancer via RECK. *Anticancer. Res.* **2018**, *38*, 2031–2035, doi:10.21873/anticanres.12442.
478. Fan, L.; Wu, Y.; Wang, J.; He, J.; Han, X. Sevoflurane inhibits the migration and invasion of colorectal cancer cells through regulating ERK/MMP-9 pathway by up-regulating miR-203. *Eur. J. Pharmacol.* **2019**, *850*, 43–52, doi:10.1016/j.ejphar.2019.01.025.
479. Lee, Y.; Kim, S.J.; Choo, J.; Heo, G.; Yoo, J.-W.; Jung, Y.; Rhee, S.H.; Im, E. miR-23a-3p is a Key Regulator of IL-17C-Induced Tumor Angiogenesis in Colorectal Cancer. *Cells* **2020**, *9*, 1363, doi:10.3390/cells9061363.
480. Bao, Y.; Chen, Z.; Guo, Y.; Feng, Y.; Li, Z.; Han, W.; Wang, J.; Zhao, W.; Jiao, Y.; Li, K.; et al. Tumor Suppressor MicroRNA-27a in Colorectal Carcinogenesis and Progression by Targeting SGPP1 and Smad2. *PLoS ONE* **2014**, *9*, e105991, doi:10.1371/journal.pone.0105991.
481. Li, H.; Zhang, H.; Lu, G.; Li, Q.; Gu, J.; Song, Y.; Gao, S.; Ding, Y. Mechanism analysis of colorectal cancer according to the microRNA expression profile. *Oncol. Lett.* **2016**, *12*, 2329–2336, doi:10.3892/ol.2016.5027.
482. Wang, Z.; Yang, J.; Di, J.; Cui, M.; Xing, J.; Wu, F.; Wu, W.; Yang, H.; Zhang, C.; Yao, Z.; et al. Downregulated USP3 mRNA functions as a competitive endogenous RNA of SMAD4 by sponging miR-224 and promotes metastasis in colorectal cancer. *Sci. Rep.* **2017**, *7*, 4281, doi:10.1038/s41598-017-04368-3.
483. Chen, E.; Li, Q.; Wang, H.; Yang, F.; Min, L.; Yang, J. MiR-92a promotes tumorigenesis of colorectal cancer, a transcriptomic and functional based study. *Biomed. Pharmacother.* **2018**, *106*, 1370–1377, doi:10.1016/j.biopha.2018.07.098.
484. Sun, W.; Wang, X.; Li, J.; You, C.; Lu, P.; Feng, H.; Kong, Y.; Zhang, H.; Liu, Y.; Jiao, R.; et al. MicroRNA-181a promotes angiogenesis in colorectal cancer by targeting SRCIN1 to promote the SRC/VEGF signaling pathway. *Cell Death Dis.* **2018**, *9*, 1–13, doi:10.1038/s41419-018-0490-4.
485. Zhang, X.; Li, X.; Tan, F.; Yu, N.; Pei, H. STAT1 Inhibits MiR-181a Expression to Suppress Colorectal Cancer Cell Proliferation Through PTEN/Akt. *J. Cell. Biochem.* **2017**, *118*, 3435–3443, doi:10.1002/jcb.26000.
486. Yu, G.; Li, H.; Wang, X.; Wu, T.; Zhu, J.; Huang, S.; Wan, Y.; Tang, J. MicroRNA-19a targets tissue factor to inhibit colon cancer cells migration and invasion. *Mol. Cell. Biochem.* **2013**, *380*, 239–247, doi:10.1007/s11010-013-1679-6.
487. Cellura, D.; Pickard, K.; Quaratino, S.; Parker, H.; Strefford, J.; Thomas, G.; Mitter, R.; Mirnezami, A.; Peake, N. miR-19–Mediated Inhibition of Transglutaminase-2 Leads to Enhanced Invasion and Metastasis in Colorectal Cancer. *Mol. Cancer Res.* **2015**, *13*, 1095–1105, doi:10.1158/1541-7786.mcr-14-0466.
488. Liu, Y.; Liu, R.; Yang, F.; Cheng, R.; Chen, X.; Cui, S.; Gu, Y.; Sun, W.; You, C.; Liu, Z.; et al. miR-19a promotes colorectal cancer proliferation and migration by targeting TIA1. *Mol. Cancer* **2017**, *16*, 1–17, doi:10.1186/s12943-017-0625-8.
489. Mastropasqua, F.; Marzano, F.; Valletti, A.; Aiello, I.; Di Tullio, G.; Morgano, A.; Liuni, S.; Ranieri, E.; Guerrini, L.; Gasparre, G.; et al. TRIM8 restores p53 tumour suppressor function by blunting N-MYC activity in chemo-resistant tumours. *Mol. Cancer* **2017**, *16*, 1–16, doi:10.1186/s12943-017-0634-7.
490. Li, T.; Gao, F.; Zhang, X.-P. miR-203 enhances chemosensitivity to 5-fluorouracil by targeting thymidylate synthase in colorectal cancer. *Oncol. Rep.* **2014**, *33*, 607–614, doi:10.3892/or.2014.3646.
491. Park, S.-Y.; Kim, H.; Yoon, S.; Bae, J.A.; Choi, S.-Y.; Jung, Y.D.; Kim, K.K. KITENIN-targeting MicroRNA-124 Suppresses Colorectal Cancer Cell Motility and Tumorigenesis. *Mol. Ther.* **2014**, *22*, 1653–1664, doi:10.1038/mt.2014.105.
